# Supplementary material for: Advance care planning in multiple sclerosis (ConCure-SM): A multicenter single-arm pilot and feasibility study
Source: PLoS One. 2025 Oct 7;20(10):e0331220. doi: 10.1371/journal.pone.0331220 (PMC12503263; doi:10.1371/journal.pone.0331220)
Supplement: S2 File — (PDF) [file pone.0331220.s002.pdf]

Fondazione I.R.C.C.S.
Istituto Neurologico Carlo Besta

Sistema Socio Sanitario
Regione Lombardia

Study Protocol

Pianificazione Condivisa delle Cure nella Sclerosi Multipla: Studio pilota (ConCure-SM Phase 2 Study)

Advance Care Planning in Multiple Sclerosis: Pilot study (ConCure-SM Phase 2 Study)

Protocol No.: FISM 2020/R-Multi/024

Version: 2

Study Design: Multi-center, prospective longitudinal mixed-methods single-arm study

Study Population: People with multiple sclerosis, their significant others, and healthcare professionals

Trial Reference Number: ISRCTN48527663

Sponsor: Fondazione IRCCS Istituto Neurologico Carlo Besta, Via Celoria 11, 20133 Milan. Italy

Principal Investigators: - Alessandra Solari MD, Unit of Neuroepidemiology
- Ludovica De Panfilis PhD, Unit of Bioethics, Azienda USL-IRCCS di Reggio Emilia, Reggio Emilia, Italy

This version of the Study Protocol contains the following changes:

1) Front page: We have added the trial reference number.
2) Section 4: we have updated the link to the ACP booklet.
3) Table 1 has been slightly revised consistently with changes # 4 and # 5 below.
4) Section 5.2.5: we have added the word ‘first’ before ‘conversation’.
5) Section 5.2.6: we have revised the administration of the QOC and QOC-SO. Originally envisaged as being self-completed, these questionnaires resulted emotionally taxing in the cognitive debriefing sub-study. Consequently, the Steering Committee decided that an independent, trained interviewer will administer the QOC and the QOC-SO in an adaptive form (the nine items on communication about EOL care will be skipped if EOL care was not discussed during the first ACP conversation).
6) Section 5.2.6: we have revised the following sentence, in view of change # 5 above: ‘In the event the pwPMS/SO have difficulties in using the trial platform, the independent interviewer will administer all the questionnaires/instruments in scheduled telephone interviews.’
7) Section 5.2.6: we have added the following statement at the end of the section to improve clarity: ‘He/she records on the platform the date, duration, participants, and mode (face to face, teleconference or on the telephone) of subsequent ACP conversations that occur during follow-up.’
8) Section 5.5.2: we have added the following statement on multiple imputation method: ‘Multiple imputation of missing values will employ Rubin’s approach’.
9) Section 8.1: we have updated the members of the Steering Committee.
10) Section 8.4: we have updated the composition of the Qualitative Analysis Panel.
11) Section 8.6: we have updated the composition of the Linguistic Validation Panel.
12) We have updated Sections 8.7 (Enrolling Centers) and 8.8 (Investigators).
13) Section 12: we have added the Fondazione Italiana Sclerosi Multipla (FISM) Grant #.
14) Appendix I: we have replaced the provisional version of the booklet with the final version.
15) Appendix II: we have slightly revised the Privacy Document of the patient and of the SO adding a section on the right of access of the data subject (articles 15-21 GDPR).
16) Information leaflets: we have slightly revised the section ‘What if you decide to participate’.

Revision # 5 described above is a major change; all the other revisions are minor changes to the Study Protocol (now 2).

Table of Contents

Section	Page
1. SUMMARY	5
2. BACKGROUND	6
3. AIMS	8
4. PREVIOUS STUDY PHASE	9
5. METHODS	10
5.1 Intervention set up	10
5.1.1 Training program	10
5.1.2 Trial platform	11
5.1.3 Meetings	11
5.2 Pilot study	12
5.2.1 Study design	12
5.2.2 Participant eligibility	13
5.2.3 Recruitment	14
5.2.4 Baseline assessment (T0)	14
5.2.5 ACP conversation	15
5.2.6 Follow-up assessments (T1, T2)	15
5.2.7 Withdraw	15
5.3 Outcome measures	16
5.3.1 ACP completion	17
5.3.2 ACP engagement	17
5.3.3 Treatment preferences	17
5.3.4 Quality of the ACP conversation	17
5.3.5 Quality of life	18
5.3.6 Caregiver burden	18
5.3.7 Mood symptoms	19
5.3.8 Safety data monitoring plan	19
5.3.9 Linguistic validation	19
5.4 Nested qualitative study	20
5.4.1 Personal semi-structured interviews	20
5.4.2 Focus group meetings	21
5.5 Data analysis	22
5.5.1 Study power	22
5.5.2 Statistics	23
5.5.3 Qualitative data	23
5.5.4 Process evaluation	24
6. EXPECTED RESULTS AND IMPACT	25
7. MEASURES TAKEN TO MINIMIZE BIAS	25
8. PANELS AND CENTERS	27
8.1 Trial Steering Committee (TSC)	27
8.2 Data Safety and Monitoring Committee (DSMC)	27
8.3 Data Management and Analysis Committee (DMAC)	27
8.4 Qualitative Analysis Panel (QAP)	27
8.5 HP Training Panel (HTP)	28
8.6 Linguistic validation Panel (LP)	28
8.7 Enrolling Centers	28
8.8 Investigators	29

9. GANTT CHART	31
10. ETHICS AND ADMINISTRATIVE CONSIDERATIONS	32
10.1 Ethical considerations	32
10.2 Ethics Committee approval	32
10.3 Subject information and informed consent	32
10.4 Confidentiality	32
10.5 Protocol amendments	33
11. STUDY MANAGEMENT AND MONITORING	33
11.1 Source documents	33
11.2 Archiving of records	33
11.3 Auditing on site	34
11.4 Use and publication of study results	34
11.5 Insurance policy	34
12. FUNDING	34
13. REFERENCES	35
14. GLOSSARY OF ABBREVIATIONS	38
15. SIGNATURE PAGE	39
APPENDIXES	
APPENDIX I: The ConCure-SM booklet (final version)	
APPENDIX II: Information leaflets and informed consent forms	

1. SUMMARY

Background. Advance care planning (ACP) is advocated to provide better care at the end-of-life (EOL) for patients suffering from chronic progressive diseases. However, ACP uptake is low, and healthcare professionals (HPs) appear hesitant to engage with these discussions.

Multiple sclerosis (MS) is the most common cause of non-traumatic disability in young adults. Axonal damage and neuronal loss underlie the progressive form of the disease, characterized by complex needs and severe disability for a variable period.

ConCure-SM is a project aimed to set up and evaluate the efficacy of an ACP intervention in people with primary or secondary progressive MS (pwPMS) in Italy. The project is based on two frameworks: a theoretical (the Shared Decision-Making model) and a methodological framework (the Medical Research Council framework for developing and evaluating complex interventions). In the first ongoing project phase, the ACP booklet was produced involving the key stakeholders: pwPMS, pwPMS' significant others (SOs), and HPs. The present study describes the second project phase.

Aim. To preliminary assess the safety and efficacy of the ACP intervention (pwPMS-physician ACP conversation using the ConCure-SM booklet) in different MS care settings in Italy.

Methods. This project phase consists of two actions. In the first action, we will: train the HP of the participating centers on ACP in MS; linguistically validate the outcome measures not available in Italian. In the second action, safety and efficacy of the intervention will be pilot tested using a six-month mixed-methods prospective study. The study will involve at least 40 pwPMS from six centers (MS centers, rehabilitation centers) across the three geographic areas of Italy. The primary outcome is completion of an advance care plan document. Secondary efficacy outcomes are the quality of communication about future medical treatment and EOL care, congruence in treatment preferences between pwPMS and their carers, mood symptoms, and caregiver burden.

A qualitative study using Normalization Process Theory (personal semi-structured interviews with purposely selected pwPMS and SOs; focus group meetings with HPs) will help understand the quantitative findings, and the challenges in implementation of the intervention in clinical practice (process evaluation). This study will inform the decision to proceed with a randomized controlled trial to assess the effect of the ACP intervention.

Key words: shared decision making; advance care planning; end-of-life care; multiple sclerosis; clinician training; complex intervention; normalization process theory.

2. BACKGROUND

With a lifetime risk of 1 in 400, multiple sclerosis (MS) is the most common cause of progressive neurological disability in young adults. Approximately 2.3 million people worldwide have MS, with Canada, USA and some European countries, including Italy, having the highest prevalence rates [Browne 2014]. Around 15% of people with MS have a primary progressive course at diagnosis, and a further 35% develop secondary progressive disease after 15 years [Filippi 2018]. A mean reduction in life expectancy by 7–14 years has been reported in people with MS, with improved figures over the last two decades [Kingwell 2012, Scalfari 2013, Lunde 2017].

Few treatment options are currently available to delay or prevent further clinical worsening of people with primary or secondary progressive MS (pwPMS), who may experience a wide range of symptoms including reduced mobility, compromised sphincter control, fatigue, impaired cognition, difficulties with swallowing and speech, and pain and sensory disturbances [Higginson 2006, Giordano 2012]. PwPMS are at risk of death from aspiration pneumonia, complications of falls and fractures, and infections [Hirst 2008, Sumelahti 2010]. Nevertheless, some pwPMS live for many years, and most die in the hospital rather than at home [Hirst 2008, Sumelahti 2010, Lunde 2017].

Advance care planning (ACP) is a process that "enables individuals who have decisional capacity to identify their values, to reflect upon the meanings and consequences of serious illness scenarios, to define goals and preferences for future medical treatment and care, and to discuss these with family and healthcare professionals (HPs)" [Rietjens 2017]. ACP differs from general medical decision-making in being based around an anticipated deterioration in the health of a patient. It includes a focus on the person's wishes and preferences for the time when they lose decisional capacity. Finally, ACP encourages discussion around end-of-life (EOL) care (a subject that is generally not considered part of health care planning, and one that can be avoided by both patients and HPs). Consistently with the shared decision-making (SDM) model [Archer 2018, Coulter 2020, Elwyn 2012], ACP involves both the patient and his/her HPs who, together, make informed decisions about the patient's (future) care. Also, the family can be involved in the process, if the patient wishes.

ACP is aiming at aligning evidence-based practice and person-centered care [Forte 2018] using a bioethical referential to identify the patient's values, preferences and desires. The planning process helps the patient to identify his/her personal values and goals, understand his/her health status, and the treatment and health care options available. Finally, it is up to the patient to determine the occurrence and content of any ACP discussion: if the patient does not wish to engage in

conversations about his/her future care, this preference should be respected. The ACP process may result in the patient choosing to write an advance care plan and to appoint a trustee (or else).

On December 22, 2017, the Italian Parliament approved the first law on EOL: “Provisions for informed consent and advance directives” (L. 219/2017; http://www.trovanorme.salute.gov.it/norme/dettaglioAtto?id=62663). This law regulates not only advance directives (AD; Article 4) and ACP (Article 5), but also a number of rights citizens have regarding healthcare issues, including the right to: be fully informed about one’s health status and to give consent (or dissent) to treatment; withhold consent to lifesaving treatments; be assisted until death; express preferences and wishes through AD. Moreover, the law states that the physician has a duty to respect the patient’s wishes. According to a recent national survey on the opinion of Italian citizens on the law, 88% (1752/2000) considered it as quite or very important, and 76% had a positive attitude towards making/registering AD or ACP [De Panfilis 2020]. Importantly, the L. 219/2017 has triggered HPs and health care authorities in promoting not only educational programs on the topic, but also programs to implement ACP in the daily clinical practice.

To optimize the alignment between patient goals and the care they receive, HPs should integrate best practices in ACP in the care of pwPMS. As from a recent guideline on palliative care in MS there is no evidence of the effects of ACP for pwPMS [Solari 2020]. However, there is some evidence from non-neurological progressive and life-threatening illnesses that ACP decreases the use of life-sustaining treatment, increases hospice/palliative care, reduces hospitalizations and increases compliance with patients’ end of life wishes [Brinkman-Stoppelenburg 2014]. Further, there is evidence that MS patients and caregivers often would like to discuss the issues of death and dying and HPs should acknowledge and encourage these discussions [Golla 2015, Golla 2016]. Often HPs leave EOL discussions until the later stages of progression in MS [Walter 2019]. Patients react in different ways on discussion of future planning: a small study showed that some MS patients made clear decisions, some undertook some planning but without a clear AD and some were still “hoping for a cure” and did not wish to look ahead [Chen 2013]. However, caregivers may be left having to take difficult decisions if no planning has taken place and this was stressful for them [McCurry 2013].

ConCure-SM is a project aimed to set up and evaluate the efficacy of an ACP intervention for pwPMS in Italy. The study focuses on HP training, and originates from a multidisciplinary panel of professionals from the *Gruppo di Studio di Bioetica e Cure Palliative* of the *Società Italiana di Neurologia*.

The SDM model described above is the theoretical framework of the project [Archer 2018, Coulter 2020, Elwyn 2012]. The Medical Research Council (MRC) framework for developing and evaluating complex interventions is the methodological framework of the project. The MRC framework has a phased approach, from a pre-clinical research phase to a final phase in which the intervention is introduced into the health service, leading to a theory-driven intervention: a "bottom up" development which guarantees to enter a phase III trial with an appropriate theory and pilot work [Craig 2008]. Further, both quantitative and qualitative methods are used and integrated within the framework, in order to better appraise the effects of the (complex) intervention both as a whole and on its components.

Our study hypotheses are that ConCure-SM intervention will produce:

- Higher completion of an ACP document.
- Increased congruence in treatment preferences between pwPMS and their carers.
- Increased quality communication about EOL care.

3. AIMS

The present study describes the second phase of the ConCure-SM project. The objectives of this pilot and feasibility study are as follows:

1. To determine how many people accept the invitation to participate in the study.
2. To determine how many participants receive the intervention.
3. To estimate recruitment and refusal rates, and 6-month follow-up rates.
4. To estimate ACP completion during the 6-month follow-up (primary study outcome).
5. To estimate occurrence of serious adverse events (SAEs) and adverse events (AEs) during the 6-month follow-up.
6. To assess, qualitatively, the acceptability of the recruitment processes, assessments, intervention delivery and secondary outcome measures with key stakeholders.
7. To measure changes in secondary outcome measures.
8. To explore the barriers and facilitators to implementing ACP in pwPMS, and the influence of the clinical setting.
9. To inform the sample size estimation for a subsequent RCT, should this be feasible.

4 PREVIOUS STUDY PHASE

Early in 2020, we translated into Italian and adapted to the Italian regulation, and to the MS context, the ACP booklet of the Health Quality & Safety Commission's New Zealand National ACP Programme (https://www.myacp.org.nz). The resulting ConCure-SM booklet in its final version (Appendix I) consists of an introduction, a 'guidance' (the odd pages in most instances) and the ACP document (the even pages) to be completed electronically by the pwPMS together with his/her referring physician. The introduction explains the concepts of advance directives and ACP according to the Italian Law 219/2017, and describes why ACP is important in MS. Ten sections follow: 'My Advance Care Plan'; 'What matters to me'; 'What worries me'; 'Why I'm making an Advance Care Plan'; 'How I make decisions'; 'If I were no longer able to make decisions: my trustee'; 'Thinking about my end of life'; 'My treatment and care choices'; 'Signatures'; 'Acronyms'. If the advance care plan document is completed, the pwPMS (and, when applicable, the pwPMS trustee) sign on pages 29 and 30 (Appendix I), which is scanned and stored, together with the completed booklet, in the (electronic) medical record, by the pwPMS, and (when applicable) the pwPMS trustee. Acceptability (contents, format, envisaged administration procedure), clarity and usefulness of the provisional has been assessed via 13 personal cognitive interviews with pwPMS, and pwPMS' SOs, and a focus group meeting (FGM) with 12 HPs (7 neurologists, 3 psychologists, one nurse and one physiotherapist). Thematic analysis is ongoing. We envisage that this project phase (Phase 1) will be completed by April 2021 (Figure).

Figure. Flow chart of the ConCure-SM project.

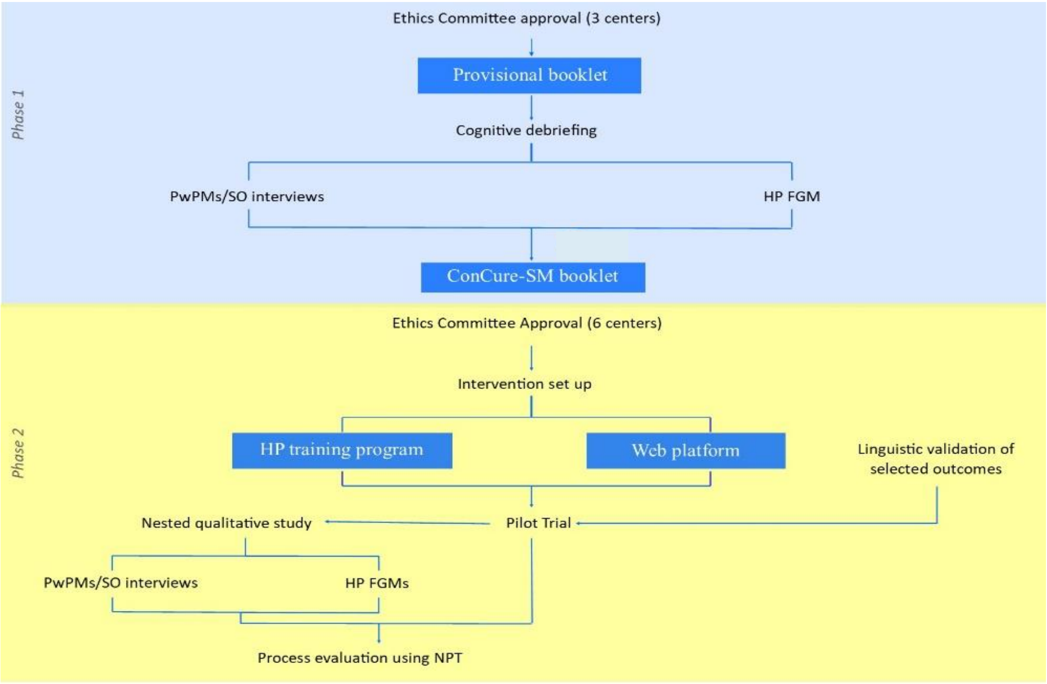

5. METHODS

5.1 Intervention set up

The intervention consists of an ACP conversation between a specially trained physician and pwPMS, using the ConCure-SM booklet (section 4). At MS centers and rehabilitation centers, physician time and space are at premium, particularly for outpatient care. For this reason, a dedicated slot (one-hour consultation room with the referring physician) will be available at each enrolling center, for intervention delivery.

5.1.1 Training program

The goal of this intervention is to prime HPs to discuss goals of care and ACP. The training program (called **Train-**ConCure-SM) will be CME accredited, residential, and last one-and-half days (12 hours). The program, devised and held by the HP Training Panel (TP), aims to:

- Improve the HP knowledge, competencies and skills in ACP based on evidence-based and up-to-date literature;
- Support and guide HPs in the ACP embedment in clinical practice;
- Regulate the communication between HPs and patient promoting an effective patient-practitioner partnership in decision-making.

The training will be interactive in style. Its residential nature and the use of role-playing exercises will ease discussion and the exchange of experiences between participants.
It will consist of the following:

- One 2.5-hour theoretical session on the clinical, ethical and statutory principles of SDM and ACP.
- Two 4-hour empirical sessions (one on each day) on conducting ACP conversations in various clinical scenarios using the ConCure-SM booklet through guided role play exercises.
- Two 45-minute self-evaluation sessions (at the beginning and at the end of the training program).

Trainees will be physicians and other HPs from the six enrolling centers. The Italian Law 219/2017 prescribes that ACP involves the patient, his/her referring physician, and (when applicable) the trustee. We decided to train also HPs other than physicians in order to promote ACP knowledge within the caring team. Each center will provide a minimum of one and no more than three

physicians, plus one HP from the following: MS nurse, therapist, psychologist, or social worker. Thus, participants will be 12-24 overall (2-4 from each center). In addition, they will be trained to the use of the trial platform during the investigators' meeting (section 5.1.4).

Trainers will be two neurologists (E Pucci, A Solari), two psychologists (K Mattarozzi, M Rimondini), one palliative care physician (S Veronese), one palliative care nurse (M Cascioli), and one bioethicist (L De Panfilis). All have consolidated experience in leading training courses and workshops on patient-clinician communication and SDM, and four (M Cascioli, L De Panfilis, E Pucci, and S Veronese) on ACP and EOL conversations. These four researchers will support physicians at the centers for issues concerning the conduction of the ACP conversation during the pilot study.

5.1.2 Trial platform

A web-based trial platform will be created containing the pseudo-anonymized trial case record form (eCRF) and the outcome measures. The platform will be ID/password protected, with dedicated accesses based on the stakeholder (pwPMS, SO, HPs, center principal investigator [PI], interviewer, data manager) and operation (completion, consultation).

5.1.3 Meetings

There will be two study meetings (teleconferences):

- The investigators' meeting will be held before enrolment starts and will last about two hours. Participants will be the Trial Steering Committee (TSC), the center PIs, and the HPs who participated in the **Train**-ConCure-SM. The aim of this meeting is to provide clear information on the study procedures, and to train HPs on the use of the trial platform.
- A second meeting will be run about two months after enrollment starts, in order to monitor possible difficulties, top up centers' motivation and provide a safe place for peer discussion on the implementation of the intervention. The meeting will last about two hours.

Additional meetings will be organized whenever needed. In addition, the study PIs and the TP will be available for inquiries about the implementation of the intervention at the participating centers.

5.2 Pilot study

5.2.1 Study design

Multi-center, pilot and feasibility mixed-methods study. It is a non-randomized study (all participants will receive the intervention).

The six centers involved are located in northern (four centers), central and southern Italy (one center each). Two of the centers are rehabilitation hospitals (one of which a research hospital), three are MS centers (two university hospitals, one research hospital) and one is a rehabilitation and MS center from a research hospital.

Recruitment will be competitive, with no pre-specified minimum number of enrolled subjects per center. The maximum number of enrolled subjects per center is 12.

There will be a baseline assessment (T0), an ACP conversation taking place within one month from the baseline assessment, and a follow-up assessment within one week of the ACP conversation (T1) and six months (T2) thereafter. The baseline and follow-up assessments will be performed via the web-based ConCure-SM platform.

Table 1. Summary of trial procedures

**Eligible pwPMS:**		► Age ≥ 18 years<br>► At least one out of seven conditions that would make ACP relevant<br>► Able to communicate in Italian<br>► Adequate cognitive and communicative ability to participate<br>► No serious psychiatric conditions<br>► No previous ACP completed
**Participant screening:**		► Confirm eligibility<br>► Obtain name/contact of SO (if applicable) and permission to contact
**Baseline assessment (T0):**	**PwMS** ➲	► General data<br>► HADS<br>► eCPS<br>► 4-item ACP Engagement<br>► MSQOL-29
	**SO** ➲	► General data<br>► ZBI
	**Physician** ➲	► PwPMS general and clinical data (EDSS [Kurtzke 1983]; Barthel Index [Mahoney 1965])<br>► Physician's general data

First ACP conversation ➲ OPTION scale (physician's competences)

**Follow-up assessment (T1):**	**PwMS** ➲	▸ HADS<br>▸ QOC (telephone, adaptive interview)<br>▸ 4-item ACP Engagement
	**SO** ➲	▸ ZBI<br>▸ QOC-SO (telephone, adaptive interview)
	**Physician** ➲	▸ QOC-Doc
**Follow-up assessment (T2):**	**PwMS** ➲	▸ HADS<br>▸ 4-item ACP Engagement<br>▸ MSQOL-29
	**SO** ➲	▸ ZBI
	**Physician** ➲	▶ PwPMS clinical/ACP update

5.2.2 Participant eligibility

We chose to focus our study population on pwPMS, as evidence suggests that patients may be less likely to engage in ACP if they perceive themselves as 'too healthy' [Schickedanz 2009].

PwPMS (in- or outpatients) will be included if they:

- Are ≥18 years of age;
- Were diagnosed with primary or secondary PMS [Lublin 1996] from one or more years before inclusion;
- Are able to communicate in Italian;
- Gave written consent;
- Have one or more of the following conditions that would make ACP relevant:
    - Express desire for ACP;
    - Raise questions about their future;
    - Talk about hastening death or medically assisted suicide;
    - High risk for death within two years using the 'Surprise Question' [Downar 2017];
    - High risk for development of severe cognitive compromise/dementia within two years;
    - High risk for development of impairments preventing communication within two years;
    - Significant suffering (e.g. uncontrolled physical symptoms, psychosocial or existential issues).

PwPMS will be excluded if they have one or more of the following:

- Severe cognitive compromise (MMSE < 19) or impairments preventing communication;
- Psychosis or other serious psychiatric conditions;
- Advance care plan document completed.

5.2.3 Recruitment

PwPMS are recruited prospectively by the ACP-trained physicians involved in their care, when the potentially eligible pwPMS attends the center for an outpatient visit or hospitalization. PwPMS who show interest in participating receive full verbal and written information about the study purpose and procedures.

5.2.4 Baseline assessment (T0)

The ACP-trained physician makes an appointment with pwPMS who provided initial verbal consent to participate in the study, and checks all eligibility criteria.

A written, signed informed consent is obtained, according to the Declaration of Helsinki and to the Good Clinical Practice (GCP) Guidelines of the EU. The informed consent is kept on file by the study personnel, and is available for inspection by regulatory authorities or authorized persons.

Then, the physician gives the pwPMS the credentials to the trial platform, so that the pwPMS completes the baseline set of questionnaires/instruments (the total assessment will last about 40 minutes). In the event the pwPMS has difficulties in using the trial platform, a phone interview is scheduled within a week with an independent, trained interviewer who will administer the questionnaires/instruments.

The ACP conversation is scheduled at the center, within three weeks. The pwPMS is invited to involve his/her SO (family member, relative, or friend, who is next of kin or is key decision maker as designated by the pwPMS and with whom the pwPMS shares his/her life). If the pwPMS agrees on involving his SO, a SO's telephone number is asked for so that the SO can be contacted by a study researcher to confirm eligibility, explain the study and obtain verbal consent. Consenting SOs receive credentials to access the trial platform and complete the baseline set of questionnaires (completion time about 15 minutes). If the SO has difficulty in using the trial platform, a phone interview is scheduled within a week with an independent, trained interviewer who will administer the questionnaires.

Finally, the physician completes the CRF via the trial platform.

Each center will collect information on the number of pwPMS and SOs approached, screened, and eligible prior to enrollment, with reasons for non-enrolment.

5.2.5 ACP conversation

The ACP conversation involves the pwPMS, the ACP-trained physician involved in his/her care and, when applicable, the SO. In addition, if the pwPMS agrees, the non-physician ACP-trained HP at the center will participate.

The first conversation takes place in a dedicated room at the center, and is audio-recorded. A one-hour slot is reserved for the conversation, and in the case a SO participates it is envisaged that there will be a session closed to the SO, followed by an open session.

About one week before the scheduled first ACP conversation, reminder emails (or phone calls) are sent to pwPMS/SOs. At the end of the first ACP conversation, the physician invites the pwPMS (when applicable the SO) to complete the T1 follow-up assessment within one week. The physician completes the QOC-Doc immediately after the ACP conversation terminates.

5.2.6 Follow-up assessments (T1, T2)

The pwPMS completes the questionnaires by one week (T1, assessment time of about 20 minutes) and six months after (T2, assessment time of about 30 minutes) the ACP conversation. The SO completes the questionnaires/instruments (T1, assessment time of about 20 minutes). All the questionnaires are self-completed using the trial platform, except the QOC and the QOC-SO. These questionnaires, originally envisaged as being self-completed, resulted emotionally taxing in the cognitive debriefing sub-study (section 5.3.9). Consequently, an independent, trained interviewer will administer the QOC and the QOC-SO in a phone interview. Before the interview, the interviewer will be notified whether, in the first ACP conversation, EOL care was discussed, or not. If not discussed, the nine QOC/QOC-SO items on communication about EOL care (section 5.3.4) will be skipped (adaptive administration).

In the event the pwPMS/SO have difficulties in using the trial platform, the independent interviewer will administer all the questionnaires/instruments in scheduled telephone interviews.

About one week before the T2 assessment, reminder emails (or phone calls) are sent to pwPMS.

The physician completes the questionnaire (T1, QOC-Doc) and the CRF using the trial platform.

He/she records on the platform the date, duration, participants, and mode (face to face, teleconference or on the telephone) of subsequent ACP conversations that occur during follow-up.

5.2.7 Withdraw

Participants (pwPMS, SOs) will be free to withdraw from the study at any time, without giving reasons and with no risk of prejudicing future care. Study personnel will make every effort to obtain, and record, information about the drop out reasons.

5.3 Outcome Measures

A range of measures will be collected to capture the full process of ACP and whether the ConCure-SM intervention has any effect on completion of an advance care plan document (primary outcome measure), congruence in treatment preferences between pwPMS and their carers, quality of patient-clinician communication, and caregiver burden. In addition, since a study-related increase in emotional burden can't be excluded, serious adverse events (SAE: admission to psychiatric ward, suicide attempt, death) will be monitored by the independent Data and Safety Monitoring Committee (DSMC). Mood symptoms will be assessed at baseline and during follow-up.

We are aware that long-term outcomes (chiefly the concordance between preferred and received EOL care and treatments) are warranted. However, the MS trajectory further challenges the collection of this outcome in the typical timeframe of a clinical trial. The assessment of this outcome in pwPMS could be feasible by focusing on the late stages of the disease only. In line with the principles of ACP, we agreed not to narrow the inclusion criteria deserving this relevant outcome to future studies.

We will use the published Italian version of the following inventories: CPS [Giordano 2008], HADS [Costantini 1999], OPTION [Goss 2007], MSQOL-29 [Rosato 2016], ZBI [Chattat 2010].

The 4-item ACP-E and the QOC inventories will be translated and culturally adapted from source language (section 5.3.8).

Table 2. Secondary outcome measures of the trial (in alphabetical order)

Scale name	*Assessor*	*Construct*	*Author*	*Italian version*	*Timing*
4-item ACP-E	Patient	ACP engagement	Sudore 2017	–	T0/T1/T2
eCPS	Patient	Role preferences	Degner 1997	Solari 2013	T0
HADS	Patient	Mood symptoms	Zigmond 1983	Costantini 1999	T0/T1/T2
MSQOL-29	Patient	Health-related QOL	Rosato 2016	Rosato 2016	T0/T2

OPTION	Third observer	SDM (physician's skills)	Elwyn 2005	Goss 2007	–
QOC	Patient	Communication quality (physician's skills)	Engelberg 2006	–	T1
QOC-Doc	Physician	Communication quality (physician's skills)	–	–	T1
QOC-SO	SO	Communication quality (physician's skills)	–	–	T1
ZBI	SO	Caregiver burden	Hérbert 2000	Chattat 2010	T0/T1/T2

5.3.1 ACP completion

Standard ACP is measured by the completion of advance care plan in the patient medical record. We will assess occurrence and signature date of advance care plan document.

5.3.2 ACP engagement

The ACP process will be assessed using the 4-item ACP engagement questionnaire. Originally developed and validated to measure the complex behavior of ACP, it consists of different versions (i.e. 55-item, 34-item, 9-item, 4-item). In this study, we will use the 4-item version which focuses on the readiness behavior change construct within the quality of life ACP domain. Responses are on a 5-point Likert scale (1 "I have never thought about it"; 2 "I have thought about it, but I am not ready to do it"; 3 "I am thinking about doing it in the next 6 months"; 4 "I am definitely planning to do it in the next 30 days"; 5 "I have already done it") [Sudore 2017].

5.3.3 Treatment preferences

CPS - The Control Preference Scale (CPS) is the most frequently used instrument to assess patient preferences for involvement in decisions about their health [Degner 2007; Kryworuchko 2008]. It consists of five ''cards'' on a board, each illustrating a different role in decision-making by means of a cartoon and short descriptive statement. In its original version, administration requires a trained examiner, who asks the patient to choose the preferred card, which is then covered up. The procedure continues (four choices) until one card is left. If the second preference is incongruent with the first (non- adjacent pairing, such as card A with card C), the test is explained again, and immediately re-administered. In the event of a further incongruence, the test is not re-administered, and a preference is not assigned. Six scores are possible based on the subject's two most preferred roles: active–active, active–collaborative, collaborative–active, collaborative–passive, passive–collaborative, and passive–passive. These scores are grouped as: active (active–active or active–collaborative), collaborative (collaborative–active or collaborative–passive), or

passive (passive–collaborative or passive–passive) [Degner 2007]. We will use the electronic Italian self-administered CPS (eCPS) [Solari 2013].

5.3.4 Quality of the ACP conversation

OPTION scale - Structured observations of audio recorded ACP conversations will be assessed using the OPTION scale. The ACP conversation will be unobtrusively audio-taped and transcribed verbatim; subsequently it will be rated by a specially trained third observer using the Observing Patient Involvement in Shared Decision Making (OPTION) scale. OPTION (http://www.glynelwyn.com/observer-option-instrument.html) is an observer-based scale that evaluates the behavior of the physician in terms of patient involvement in decision-making [Elwyn 2005]. It consists of 12 items, each rated on a five-point Likert scale ranging from 0 (behavior not observed) to 4 (behavior observed to high standard). A total score (range 0–48) is obtained by adding the scores of each item. OPTION has been validated in seven languages, including Italian [Goss 2007].

QOC, QOC-SO and QOC-Doc - After the ACP conversation, pwPMS will complete the Quality of Communication Questionnaire (QOC) [Engelberg 2006]; SOs will complete the SO version, and physicians the physician version (last two items) of the QOC. Developed from qualitative studies with patients, families, and clinicians, the QOC consists of 19 items measuring general communication (ten items) and communication about EOL care (nine items), each rated on a scale from 0 ('very worst I can imagine'/'not at all') to 10 ('very best I can imagine'/'extremely'), or identified as something the clinician did not do. The 0/10 ratings are recoded to 1/11, with 0 imputed for 'did not do ('http://depts.washington.edu/eolcare/products/instruments/). A SO's version of the inventory (QOC-SO), and a physician's version (QOC-Doc, made of the last two items only) will be also devised for this study.

5.3.5 Quality of life

The 29-item Multiple Sclerosis Quality of Life (MSQOL-29) is the shortened form of the MSQOL-54 [Rosato 2016; Rosato 2019], one of the most used HRQOL inventories in MS. It includes 25 items forming 7 subscales and 4 single items, and one filter question for 3 'sexual function' items. We will use the electronic version (eMSQOL-29) of the questionnaire [Rosato 2019].

5.3.6 Caregiver burden

The Zarit Burden Interview (ZBI) [Hérbert 2000] is a 22-item self-report measure of subjective burden among caregivers. It addresses functional and behavioral impairments as well as the home care circumstances. Items are scored on a 5-point Likert scale ranging from 0 (never) to 4 (nearly always present). A total 0 (low burden) to 88 (high burden) score is obtained by summing item responses. We will use the Italian ZBI version [Chattat 2010].

5.3.7 Mood symptoms

HADS - The Hospital Anxiety and Depression Scale (HADS) is a self-assessed questionnaire consisting of 14 multiple-choice (0–3 Likert scale) items probing symptoms of anxiety (7 items) and depression (7 items). HADS anxiety (HADS-A) and depression (HADS-D) scores range from 0 (no symptoms) to 21 (most severe symptoms) [Zigmond 1983]. We choose HADS because it is a screening tool with robust psychometric properties, and has been validated in several languages, including Italian [Costantini 1999]. Furthermore, by omitting items assessing somatic symptoms, and thereby limiting false positive findings, the scale is suitable for use in persons with medical conditions [Herrmann 1997]. A cutoff score of 8 or above was recommended for MS patients, since it was found to be an accurate indicator of major depression (90% sensitivity, 87% specificity) and generalized anxiety disorder (88.5% sensitivity; 81% specificity) in this population [Honarmand 2009]. We will use the Italian version of the HADS [Costantini 1999].

5.3.8 Safety data monitoring plan

A thorough safety data monitoring plan will be developed and implemented in order to monitor participant safety, as well as data integrity. Adverse events (AEs) will be recorded by the site and reported to the PI immediately.

For the ConCure-SM Phase 2 study, a Serious Adverse Event (SAE) will be defined as an emotional breakdown resulting in hospitalization, suicidal attempt, or death. AEs will include the following: a) any contact to the patient referring physician due to the occurrence of emotional problems during the study; b) an increase of ≥ 20% in the HADS Anxiety or/and depression score [Puhan 2008]. AEs will be monitored to ensure that the AE was caused by the study intervention, and not some coincidental issue. AEs will be collected and reported to the study PI as well as the DMSC.

5.3.9 Linguistic validation

The 4-item ACP-E and the QOC inventories will be translated and culturally adapted from source language to Italian following accepted guidelines [Guillemin 1993; Wild 2009]. The main steps in this process are the following:

Forward translation - The aim of this step is to produce a version semantically and conceptually as close as possible to the original. Two qualified translators, both living in the target country, will produce two independent translations. A panel consisting of the translators and HPs will review the forward translations and a consensus version will be produced.

Backward translation - The consensus translation generated in step 1 will be independently translated back into the source language by a third qualified translator, living in the target country. The backward translation will be produced without access to the original version and without consulting the other translators.

Consensus meeting - At a meeting between those participating in step 1 and the backward translator, the backward translation will be compared with the original, and further refinements to the target version will be made. Differences will be resolved by discussion. After translation into the target language, the instrument will be administered to and tested (debriefed) involving 5 to 10 users. Results of cross-cultural testing will be discussed in a joint meeting.

5.4 Nested qualitative study

Our referral framework was the mixed methods approach which, by integrating the quantitative findings with the qualitative findings, aims to provide a more comprehensive picture of the intervention than either method can do [Craig 2008]. Quantitative and qualitative data will be collected in parallel and then analyzed and integrated using Normalization Process Theory (NPT). We will perform semi-structured one-on-one interviews with pwPMS and SOs chosen using a maximum variation strategy, and FGMs of physicians involved in intervention delivery and other HPs from the center (Figure). For the pwPMS and the SOs the personal semi-structured interviews were considered most appropriate to limit interview burden and hopefully make it easier for participants to express their feelings, and recount their experiences of the intervention. For the patient referring physicians and the other HPs we chose the FGMs as they promote interaction and exchange of ideas. A minimum of 10 interviews (five with pwPMS and five with SOs) and two FGMs will be held, the final number depending on the achievement of 'data saturation' [Denzin 2000].

We will use NPT to determine if, and in what ways, the ConCure-SM intervention can be successfully 'normalized' (embedded) into clinical practice. Further, we will use qualitative data to identify required modifications and to develop practical strategies for enabling and sustaining the ConCure-SM intervention delivery in clinical settings.

5.4.1 Personal semi-structured interviews

The interviews will take place within two months of trial completion, and last no more than an hour. We plan to conduct a minimum of five pwPMS and five SOs interviews. Data will be analyzed after a set of two to three interviews and used to decide the characteristics of the next interviewee, revise the interview guide (if necessary), and indicate when recruitment should stop because of adequate evidence of 'data saturation' (a point where data collected from the conducting additional interviews became redundant, adding no more to the understanding of the study phenomenon) [Denzin 2000].

The 'maximum-variation' selection strategy will be designed to include participants with varying characteristics and levels of outcomes. To reduce social desirability response bias, the interviews will be conducted by researchers not involved in the ConCure-SM intervention delivery. The interview will be audio recorded, transcribed, and analyzed using thematic analysis, guided [Braun 2006]. Participant consent for the interviews will be sought separately. The interviews will provide important feedback on participant perception of the quality of the intervention provided, and will serve as a process measure. Insights from this qualitative analysis will serve to inform fine-grain intervention refinement.

Before starting, interviewees will be informed of study aims and requirements, and provide written consent. The interviewer will then explain that the aim of the interview was to obtain participant feedback on experience of the pilot study and stress that positive and negative experiences of, and feelings about, the intervention are welcome. Participants will be assured that the interviews are confidential, and that the audio recordings and subsequent transcripts will be fully anonymized. The interviewer will then pose each question in turn, neutrally (so as to not suggest any particular reply) and in an open-ended fashion (to allow many possible replies). As each question is discussed, follow-up questions will clarify and explore participant responses. Participants will be also encouraged to elaborate on any pertinent themes or views that emerge. The interviewer will note any potentially informative non-verbal gestures. At the end of the interview, the interviewer will verbally summarize the key points and ask the participant if the summary is full and correct.

5.4.2 Focus group meetings (FGMs)

Two FGMs, each with 6-10 physicians who delivered the intervention and HPs from the participating centers will be conducted. Each meeting will last about 2 hours, and will be conducted by two psychologists specifically trained in qualitative research. The aim is to collect insights and living

experiences about the intervention and to identify possible barriers to its implementation; they will provide important feedback on the intervention and on factors that can enable its implementation and adoption. For this reason, HPs other than the physicians involved in the ACP conversation will be involved.

All participants will provide written informed consent prior to participating. The meetings (teleconferences) will be audio-recorded and transcribed verbatim. They will be conducted by a single facilitator, whose job is to engage all participants, promote exchange, moderate conflicts, ensure that all pre-specified topics will be adequately covered, and allow exploration of any pertinent issues that arise. He/she will first explain the purpose of the meeting and ask participants to introduce themselves. He/she will then introduce each topic in turn, in an open-ended fashion. At any point the facilitator can probe for further information and ask follow-up questions to stimulate further discussion. After all pre-specified topics are fully discussed, the facilitator will summarize the main points, and ask for further feedback and whether all concerns have been fully aired. The co-moderator will take notes and oversee the audio recording. Subsequently, the facilitator will produce a report from the audio recordings/transcript and his/her field notes, which will be submitted to participants for review (respondent validation).

5.5 Data analysis

5.5.1 Study power

As this is a pilot and feasibility study, a formal sample size calculation is not required. We aim to recruit at least 40 pwPMS from six centers to assess feasibility across a diverse range of participants including those with different care needs and living conditions. There are no data available on the occurrence of ACP in pwPMS: by hypothesizing a proportion in the pwPMS population of 10%, a sample size of 35 subjects achieves a power of 90%, assuming a type I error of 5%, to detect a proportion of ACP documentation of 30% (Table 2). By hypothesizing a proportion in the pwPMS population of 8%, a sample size of 35 subjects achieves a power of 95%, assuming a type I error of 5%, to detect a proportion of ACP documentation of 30% (Table). By adding 15% of drop outs or incomplete data, 40 pwPMS should be recruited.

Table 3. Estimated sample size for one-sample comparison of proportion to hypothesized value. Ho is the value in the population, Hi is the alternative value.

Ho	*Hi*	*Alpha (two-sided)*	*Power*	*Estimated required sample size (n)*

0.10	0.30	0.05	0.90	35
0.08	0.30	0.05	0.95	35

5.5.2 Statistics

Descriptive statistics will be calculated for general and clinical variables. Specifically, continuous variables will be summarized by their mean and SD, or median and interquartile range; categorical variables will be summarized as numbers and percentages.

Categorical variables will be compared using the chi-squared test. The normality assumption of continuous variables will be tested with the Shapiro-Wilk test. Depending on data distribution, between-group comparisons will be carried out using either the two-sided unpaired t-test or the Wilcoxon two sided two sample test; within-group comparisons will be carried out using either the paired t-test or the Wilcoxon signed-rank test; correlations will be computed using Pearson's or Spearman's coefficients.

Our primary end-point is the proportion of pwPMS completing an ACP during the six-month period. Change in the secondary outcome measures will be also calculated. In addition, we will calculate the following feasibility outcomes:

- Recruitment rate (enrollment per month; reasons for non-eligibility, non-enrollment).
- Retention rate (proportion completing the intervention and study follow-up).
- Missing data (proportion fully completed, for each scale, at each time point).

Data will be analyzed according to the intention-to-treat principle. Multiple imputation of missing values will employ Rubin's approach. A p-value less than 0.05 will be considered statistically significant. No correction for multiple comparisons will be applied. All analyses will be performed using STATA 16 (College Station, Texas 77845 USA). Assumptions in determining the sample size of the main trial will be checked.

5.5.3 Qualitative data

Interviews and FGMs will be audio-recorded and transcribed verbatim. Data analysis will be conducted by three researchers with experience in qualitative research.

Researchers will analyze personal interviews and FGM data using thematic analysis, with interpretation guided by NPT components (coherence; cognitive participation; collective action; reflexive monitoring). Data will be triangulated across sources.

The analytical stages can be summarized as follows [Braun 2006]:

- Each researcher will read the transcriptions and write comments and initial thoughts in a memo.
- Each researcher will extract portions of the text individually and then share their work to reach an initial agreement. During this stage, they will conduct the thematic analysis inductively providing their insights.
- Researchers will independently review themes and allocate portions of the text to the newly reconfigured themes.
- Together, they will re-define themes and re-name them to achieve internal consistency.
- One researcher will extract from the interviews and draft the final report, which will be checked and amended by the other two.

5.5.4 Process evaluation

We will follow the MRC guidance on process evaluation [Moore], which describes three components using a mixed-methods approach: implementation or delivery; mechanisms of impact; contextual factors. Process evaluation of complex interventions usually requires a combination of quantitative and qualitative methods: at the feasibility and piloting stage, basic quantitative measures of implementation may be combined with in-depth qualitative data to provide detailed understandings of intervention functioning on a small scale [Moore 2014] (Figure).

Quantitative measures will include structured observations of audio recorded ACP conversations. These will be used to examine aspects of fidelity (such as consistency with SDM principles), and dose (the duration of conversations).

Qualitative methods will be used to investigate mechanisms of impact and contextual factors, using NPT [Murray 2010, May 2018]. NPT identifies four essential determinants of 'normalising' complex interventions into common practice: *coherence* (the extent to which an intervention is understood as being meaningful, achievable and valuable); *cognitive participation* (the engagement of HPs necessary to deliver the intervention); *collective action* (the work that brings the intervention into use); and *reflexive monitoring* (the on-going process of adjusting the intervention to keep it in place) [May 2018]. These components are considered to be dynamic and interact within the wider context of the intervention, such as existing organizational structures and procedures [May 2018].

6 EXPECTED RESULTS AND IMPACT

Research – The safety and efficacy of the ConCure-SM intervention will be preliminary assessed, using mixed methods approach. A protocol for a phase III trial will be developed if this trial's findings demonstrate that: a) the intervention is acceptable to > 70% of participants (pwPMS, SOs, and physicians); > 50% of eligible pwPMS are recruited; > 70% of recruited pwMS are retained; > 25% of recruited pwMS complete an ACP document.

Deliverables - This study will produce an HP training program and booklet that are specific for ACP in pwPMS, devised with the contribution of the key stakeholders. The electronic format will ease the incorporation of the ACP document (and its updates) in the electronic medical record, that is currently available in some Italian regions and hopefully will be soon available all over Italy.

Use in other conditions – The ConCure-SM intervention can be adapted for use in other neurological and non-neurological conditions for which consolidated ACP interventions are not available.

7 MEASURES TAKEN TO MINIMIZE BIAS

Co-production - A strong relationship exists between the engagement of stakeholders in the planning and design of a study and the subsequent quality and outcomes [Washington 2011]. The ConCure-SM resource was constructed involving the authors of the original booklet, and all the key stakeholders, in a co-production approach. The TSC is formed by the same clinicians, researchers, patient representative, and patient expert. This in order to assure all the necessary expertise to resource's development, and testing.

Safety and quality assurance - The study follows the CONSORT guidance for trials on social and psychological interventions (CONSORT-SPI 2018) [Grant 2018], as many of the guidance items (excluding items that are specific to the randomization nature of the study) are relevant for reporting other types of pilot and feasibility studies [Lancaster 2019]. This includes the devise of the study protocol following the SPIRIT guidance [Calvert 2018], protocol's publication, and the trial public registration (ISRCTN registry).

The consolidated criteria for reporting qualitative research will guide the presentation of findings in the study reports and publications [Tong 2007, O'Brien 2014].

All study personnel will be trained to conform to GCP regulation.

Electronic version of the study questionnaires/inventories will be used to ensure the high quality of the entered data.

Finally, an independent DSMC will monitor and supervise the progress of the study, and the safety data.

Intervention focus – The ConCure-SM intervention specifically trains MS neurologists and other HPs in ACP. We considered to target pwPMS instead, or both parties, however at this stage of the project we preferred to have a clear focus on enhancing HP competencies. A multiple-component approach that targets clinicians and pwPMS simultaneously has been suggested in other disciplines [Schichtel 2019], and will be considered in the next project phase.

Generalizability - Participants (pwPMS, SOs, and HPs) will be enrolled from university hospitals, research hospitals and clinical centers from the different areas of Italy. Personal, semi-structured interviews and FGMs will be run via video teleconference, which will ease participation of pwPMS with severe disability and SOs with caregiving commitments, as well as HPs. In the event potentially revealing pwPMS and/or SOs have no access to internet using personal computer or other devices, such participants will be interviewed on the telephone. The training program will be face-to-face as a substantial part of it is experiential, via role play exercises. The investigators meetings, and the meetings of the study committees will be held via teleconferences.

8 PANELS AND CENTERS

8.1 Trial Steering Committee (TSC)

The TSC is the executive body for the study. Members are from the *Gruppo di Studio di Bioetica e Cure Palliative* of the *Società Italiana di Neurologia* (M Cascioli, L De Panfilis, MG Grasso, A Giordano, A Lugaresi, E Pucci, A Solari, C Solaro, S Veronese), from the *National ACP programme for New Zealand* (L Manson), from patient associations (M Bruzzone, P Kruger), and MS neurologists (A Gajofatto, F Patti).

8.2 Data Safety and Monitoring Committee (DSMC)

The independent DSMC has been established to: (1) oversee the progress of the pilot study and the safety data, and ensure that it is conducted, recorded, and reported in accordance with the protocol, GCP, and the applicable regulatory requirement(s); (2) monitor and supervise the progress of the pilot study, and the safety data. Members are: K Brazil, B Farsides, L Orsi, C Peruselli, and D Oliver (Chair). The DSMC is scheduled to meet (teleconference) before enrollment starts, at the end of the enrollment, and at the end of the follow-up, and depending on the needs of the trial. One week prior to each teleconference, the trial PI will send each DSMC member a report with trial data (overall and by site) such as recruitment rates, reasons for exclusion, reason for drop out, plus other information if needed. The DSMC should report in writing to the TSC, usually within 3 weeks after the teleconference.

8.3 Data Management and Analysis Committee (DMAC)

The DMAC is responsible for data entry, quality assurance, and the statistical analyses. Members are M Farinotti (data manager) and A Giordano. DMAC will be in charge of the data protection to respond to the European and Italian law on privacy and data storage and conservation.

8.4 Qualitative Analysis Panel (QAP)

The QAP devised the design, procedures and analysis plan of the qualitative study. QAP members will conduct the personal interviews and the FGMs, and the analysis. Members are: M Cascioli, L De Panfilis, L Ghirotto, K Mattarozzi, M Perin, and S Veronese.

8.5 HP Training Panel (HTP)

The HTP devised the HP training program. HTP members will have responsibility of conducting the residential program, and revise it based on training findings. Members are: M Cascioli, L De Panfilis, K Mattarozzi, E Pucci, M Rimondini, A Solari, and S Veronese.

8.6 Linguistic validation Panel (LP)

The LP was appointed to translate and adapt the outcome measures not available in Italian. Members are M Farinotti, A Giordano, P Kruger, A Solari, S Veronese and three independent translators (section 5.3.8).

8.7 Enrolling Centers

Centers that will enroll participants for the pilot study will be the following (from North to South of Italy):

1. Department of Neuroscience, Biomedicine and Movement Sciences, University of Verona; Unit of Neurology, Borgo Roma Hospital, Azienda Ospedaliera Universitaria Integrata Verona, Verona, Italy: A Gajofatto (Center PI), F Gobbin, R Orlandi
2. Department of Rehabilitation M. L. Novarese Hospital, Moncrivello, Vercelli: C Solaro (Center PI), E Grange
3. Azienda USL-IRCCS di Reggio Emilia, Reggio Emilia: L De Panfilis, S Montepietra (Center PIs), F Sireci
4. UOSI Riabilitazione Sclerosi Multipla, IRCCS Istituto delle Scienze Neurologiche di Bologna; Dipartimento di Scienze Biomediche e Neuromotorie, Università di Bologna, Bologna: A Lugaresi, L Sabbatini (Center PIs), C Scandellari, E Ferriani
5. Fondazione IRCCS Santa Lucia, Roma: MG Grasso (Center PI), G Presicce
6. University Hospital Policlinico Vittorio Emanuele, Catania: F Patti (Center PI), CG Chisari, S Toscano

8.8 Investigators

Kevin Brazil PhD	School of Nursing and Midwifery, Queen's University of Belfast, Belfast, Northern Ireland, UK
Michela Bruzzone MSc	The Italian Multiple Sclerosis Society, Genoa, Italy
Marta Cascioli BSc	Hospice "La Torre sul Colle", Usl Umbria 2, Spoleto (PG), Italy
Clara Grazia Chisari MD	University Hospital Policlinico Vittorio Emanuele, Catania, Italy
Ludovica De Panfilis MSc	Unit of Bioethics, Azienda USL-IRCCS di Reggio Emilia, Reggio Emilia, Italy
Mariangela Farinotti	Unit of Neuroepidemiology, Fondazione IRCCS Istituto Neurologico Carlo Besta, Milan, Italy
Bobbie Farsides PhD	Brighton & Sussex Medical School, Falmer, Brighton, United Kingdom
Elisa Ferriani MSc	UOSI Riabilitazione Sclerosi Multipla, IRCCS Istituto delle Scienze Neurologiche di Bologna; Dipartimento di Scienze Biomediche e Neuromotorie, Università di Bologna, Bologna, Italy
Alberto Gajofatto MD, PhD	Department of Neuroscience, Biomedicine and Movement Sciences, University of Verona; Unit of Neurology, Borgo Roma Hospital, Azienda Ospedaliera Universitaria Integrata Verona, Verona, Italy
Luca Ghirotto PhD	Qualitative Research Unit, Azienda USL-IRCCS di Reggio Emilia, Reggio Emilia, Italy
Andrea Giordano PhD	Unit of Neuroepidemiology, Fondazione IRCCS Istituto Neurologico Carlo Besta, Milan, Italy
Francesca Gobbin MD	Department of Neuroscience, Biomedicine and Movement Sciences, University of Verona; Unit of Neurology, Borgo Roma Hospital, Azienda Ospedaliera Universitaria Integrata Verona, Verona, Italy
Enrica Grange MSc	Department of Rehabilitation M. L. Novarese Hospital, Moncrivello, Vercelli, Italy
Maria Grazia Grasso MD, PhD	Multiple Sclerosis Unit, IRCCS S. Lucia Foundation, Rome, Italy
Paola Kruger	The European Patients' Academy (EUPATI), Rome, Italy
Alessandra Lugaresi MD, PhD	UOSI Riabilitazione Sclerosi Multipla, IRCCS Istituto delle Scienze Neurologiche di Bologna; Dipartimento di Scienze Biomediche e Neuromotorie, Università di Bologna, Bologna, Italy
Leigh Manson	Health Quality & Safety Commission New Zeland, Nelson, New Zealand
Katia Mattarozzi MSc, PhD	Department of Experimental, Diagnostic and Specialistic Medicine, School of Medicine, Alma Mater Studiorum University of Bologna, Italy
Sara Montepietra MD	Multiple Sclerosis Center, Azienda USL-IRCCS di Reggio Emilia, Reggio Emilia, Italy
David Oliver PhD	The Tizard Centre, University of Kent, Canterbury, UK
Riccardo Orlandi MD	Department of Neuroscience, Biomedicine and Movement Sciences, University of Verona; Unit of Neurology, Borgo Roma Hospital, Azienda Ospedaliera Universitaria Integrata Verona, Verona, Italy
Luciano Orsi MD	The Italian Society of Palliative Care, Milan, Italy
Francesco Patti MD	University Hospital Policlinico Vittorio Emanuele, Catania, Italy
Marta Perin MSc	Unit of Bioethics, Azienda USL-IRCCS di Reggio Emilia, Reggio Emilia, Italy
Carlo Peruselli MD	The Italian Society of Palliative Care, Milan, Italy

Giorgia Presicce MD	Multiple Sclerosis Unit, IRCCS S. Lucia Foundation, Rome, Italy
Eugenio Pucci MD	UOC Neurologia, ASUR Marche, Fermo, Italy
Michela Rimondini MSc, PhD	Section of Clinical Psychology, Department of Neuroscience, Biomedicine and Movement Sciences, University of Verona, Policlinico G.B. Rossi, Verona, Italy
Loredana Sabbatini MD	UOSI Riabilitazione Sclerosi Multipla, IRCCS Istituto delle Scienze Neurologiche di Bologna; Dipartimento di Scienze Biomediche e Neuromotorie, Università di Bologna, Bologna, Italy
Cinzia Scandellari MD	UOSI Riabilitazione Sclerosi Multipla, IRCCS Istituto delle Scienze Neurologiche di Bologna; Dipartimento di Scienze Biomediche e Neuromotorie, Università di Bologna, Bologna, Italy
Francesca Sireci MSc	Multiple Sclerosis Center, Azienda USL-IRCCS di Reggio Emilia, Reggio Emilia, Italy
Alessandra Solari MD	Unit of Neuroepidemiology, Fondazione IRCCS Istituto Neurologico Carlo Besta, Milan, Italy
Claudio Solaro MD	Department of Rehabilitation M. L. Novarese Hospital, Moncrivello, Vercelli, Italy
Simona Toscano MD	University Hospital Policlinico Vittorio Emanuele, Catania, Italy
Simone Veronese MD, PhD	Fondazione FARO, Turin, Italy

9 GANTT Chart

The study will be completed in two years. Each activity is reported (and detailed) in the chart below.

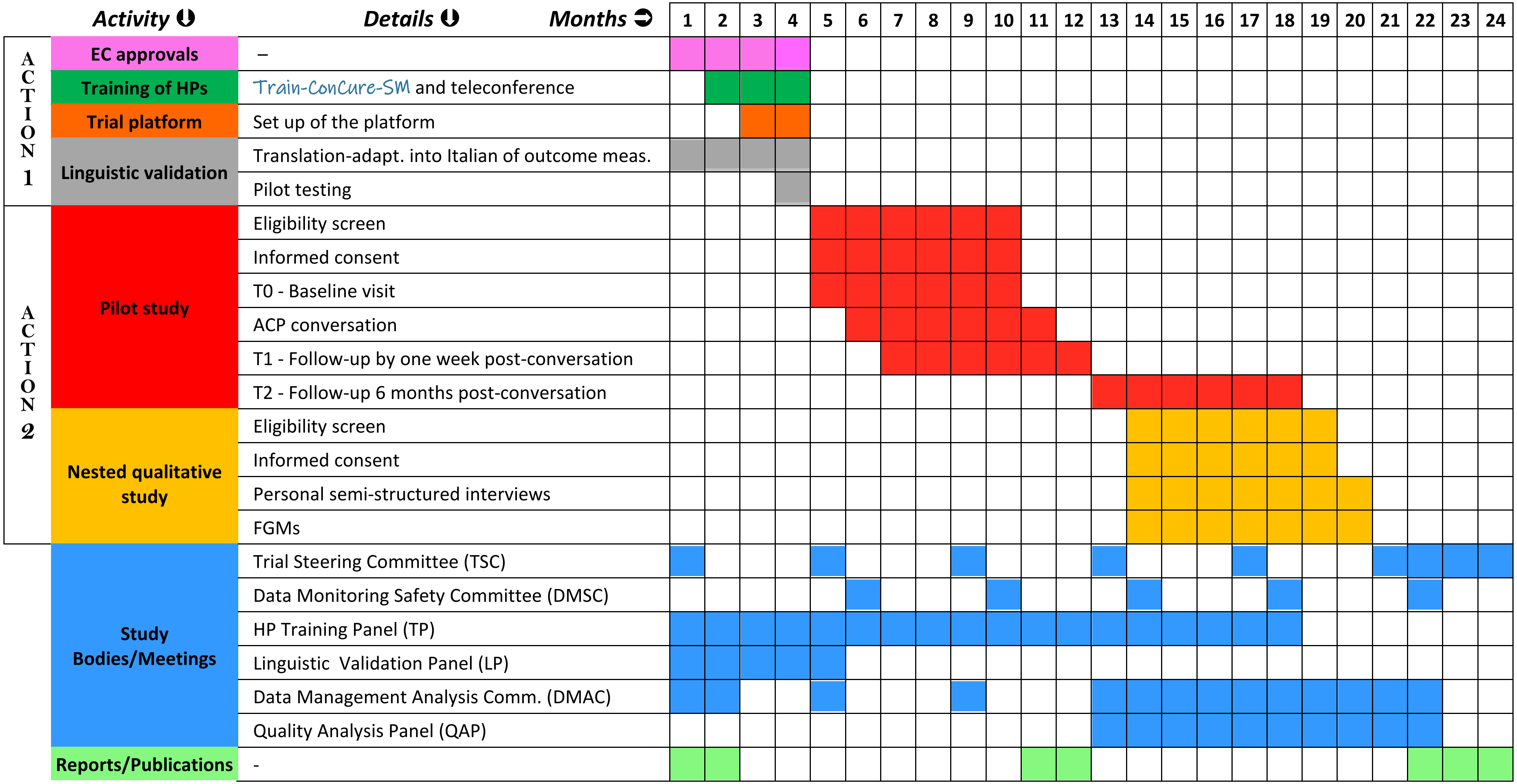

10 ETHICS AND ADMINISTRATIVE CONSIDERATIONS

10.1 Ethical Considerations

This clinical study was designed and shall be implemented and reported in accordance with the International Council for Harmonisation of Technical Requirements for Pharmaceuticals for Human Use (ICH) Guidelines for GCP, with applicable local regulations, and with the ethical principles laid down in the Declaration of Helsinki.

10.2 Ethics Committee Approval

The protocol, Subject Information Sheet, Informed Consent Form must be reviewed and approved by an appropriately constituted Ethics Committee (EC), as required in chapter 3 of the ICH E6 Guideline. Written EC approval must be obtained by the Sponsor prior to shipment of study agent or subject enrolment.

10.3 Subject Information and Informed Consent

Eligible subjects may only be included in the study after providing written (witnessed, where required by law or regulation), EC-approved informed consent, or, if incapable of doing so, after such consent has been provided by a legally acceptable representative of the subject. In cases where the subject's representative gives consent, the subject should be informed about the study to the extent possible given his/her understanding. If the subject is capable of doing so, he/she should indicate assent by personally signing and dating the written informed consent document or a separate assent form. Informed consent must be obtained before conducting any study-specific procedures (i.e. all of the procedures described in the protocol). The process of obtaining informed consent should be documented in the subject source documents. No study procedure can be performed before the written informed consent has been provided.

10.4 Confidentiality

The investigator must ensure participant anonymity. On database and other documents, participants must not be identified by name but by patient number and initials. The investigator must keep a separate log of participants' codes, names and addresses, and signed informed consent forms, all of which must be kept strictly confidential.

Patient medical information obtained by this study is confidential and may only be disclosed to third parties as permitted by the Informed Consent Form (or separate authorization for use and disclosure of personal health information) signed by the patient, unless permitted or required by law. Medical information may be given to a pwPMS personal physician or other appropriate medical personnel responsible for the pwPMS welfare, for treatment purposes. Data generated by this study must be available for inspection upon request by representatives of the national and local health authorities, monitors, representatives, and collaborators, and the EC for each study site, as appropriate.

10.5 Protocol Amendments

Any protocol amendments will be prepared by the PI. Protocol amendments will be submitted to the EC and to regulatory authorities in accordance with local regulatory requirements. Approval must be obtained from the EC and regulatory authorities (as locally required) before implementation of any changes, except for changes necessary to eliminate an immediate hazard to patients or changes that involve logistical or administrative aspects only (e.g. change in monitor or contact information).

11 STUDY MANAGEMENT AND MONITORING

11.1 Source documents

Source Documents are defined as original documents, data and records. These may include hospital records, medical records / outpatient data, data recorded from automated instruments, etc. Investigators should conserve all the source documents as required in the study protocol for at least two years after the end of the study.

11.2 Archiving of records

The investigator is responsible for recording and storing the essential documents of the study, according to what / and for the time required by law and by GCP. The Investigator must maintain adequate and accurate records to enable the conduct of the study to be fully documented, including but not limited to the protocol, protocol amendments, Informed Consent Forms, and documentation of EC and governmental approval.

11.3 Auditing on site

In the event that the investigator will be contacted by the Competent Authority in relation to this study, he or she will be required to immediately notify the Sponsor. The investigator must be available to respond to requests and queries by inspectors during the audit process. The investigator must provide the Sponsor copies of all correspondence that may affect the revision of the current study.

11.4 Use and Publication of Study Results

The results of the study may be presented during scientific symposia or published in a scientific journal only after review and written approval by the involved parties in full respect of the privacy of the participating subjects.

11.5 Insurance Policy

Each of the participating centers has an adequate insurance policy to cover possible damages emerging from this study.

12. FUNDING

The present protocol has been funded by the Fondazione Italiana Sclerosi Multipla, FISM (Grant # FISM 2020/R-Multi/024 to A Solari).

13 REFERENCES

Archer J, Stevenson L, Coulter A, Breen AM. Connecting patient experience, leadership, and the importance of involvement, information, and empathy in the care process. Healthc Manage Forum 2018; 31(6): 252-5.

Braun V, Clarke V. Using thematic analysis in psychology. Qual Res Psychol 2006; 3:77–101.

Brinkman-Stoppelenburg A, Rietjens JAC, van der Heide A. The effects of advance care planning on end-of-life care: A systematic review. Palliat Med 2014; 28(8): 1000–25.

Browne P, Chandraratna D, Angood C, et al. Atlas of Multiple Sclerosis 2013: A growing global problem with widespread inequity. Neurology. 2014; 83(11): 1022-4.

Calvert M, Kyte D, Mercieca-Bebber R, Slade A, Chan A-W, King MT. Guidelines for Inclusion of Patient-Reported Outcomes in Clinical Trial Protocols: The SPIRIT-PRO Extension. JAMA 2018;319(5):483–494.

Chattat R, Cortesi V, Izzicupo F, et al. The Italian version of the Zarit Burden Interview: a validation study. Int Psychogeriatr 2010; 16: 1-9.

Chen H, Habermann B. Ready or not: planning for health declines in couples with advanced multiple sclerosis. J Neurosci Nurs 2013; 45: 38–43.

Coulter A, Collins A. Making shared decision-making a reality. London, United Kingdom: King's Fund. 2011. Available at: https://www.kingsfund.org.uk/publications/making-shared-decision-making-reality. Accessed March 14, 2020.

Craig P, Dieppe P, Macintyre S, et al. Medical Research Council Guidance. Developing and evaluating complex interventions: the new Medical Research Council guidance. BMJ 2008; 337: a1655.

Costantini M, Musso P, Viterbori F, et al. Detecting psychological distress in cancer patients: validity of the Italian version of the Hospital Anxiety and Depression Scale. Support Care Cancer 1999; 7: 121–7.

Degner LF, Sloan JA, Venkatesh P. The control preference scale. Can J Nurs Res 1997; 29: 21–43.

Denzin NK, Lincoln YS. Handbook of qualitative research. London, UK: Sage Publications; 2000.

Downar J, Goldman R, Pinto R, Englesakis M, Adhikari NKJ. The "surprise question" for predicting death in seriously ill patients: a systematic review and meta-analysis. CMAJ 2017; 189 (13): E484–93.

De Panfilis L, Giorgi Rossi P, Mazzini E, et al. Knowledge, opinion and attitude about the Italian law on Advance Directives: a population-based survey. J Pain Symptom Manage 2020:S0885-3924(20)30561-3.

Elwyn G, Hutchings H, Edwards A, et al. The OPTION scale: measuring the extent that clinicians involve patients in decision-making tasks. Health Expect 2005; 8: 34–42.

Elwyn G, Frosch D, Thomson R, et al. Shared decision making: a model for clinical practice. J Gen Intern Med 2012; 27: 1361-7.

Engelberg R, Downey L, Curtis JR. Psychometric characteristics of a quality of communication questionnaire assessing communication about end-of-life care.J Palliat Med. 2006;9(5):1086-98.

Filippi M, Bar-Or A, Piehl F, et al. Multiple Sclerosis. Nat Rev Dis Primers 2018; 4: 43.

Forte DN, Kawai F, Cohen C. A bioethical framework to guide the decision-making process in the care of seriously ill patients, BMC Medical Ethics (2018) 19: 78.

Giordano A, Mattarozzi K, Pucci E, Leone M, Casini F, Colimedaglia L, et al. Participation in medical decisionmaking: Attitudes of Italians with multiple sclerosis. J Neurol Sci 2008; 275: 86–91.

Giordano A, Ferrari G, Radice D, et al. on behalf of the POSMOS study. Health-related quality of life and depressive symptoms in significant others of people with multiple sclerosis: A community study. Eur J Neurol 2012; 19: 847854.

Golla H, Galushko M, Strupp J, et al. Patients feeling severely affected by multiple sclerosis: addressing death and dying. Journal of Death & Dying 2016; 74(2): 275–91.

Golla H, Mammeas S, Galushko M, Pfaff H, Voltz R. Unmet needs of caregivers of severely affected multiple sclerosis patients: A qualitative study. Palliat Support Care 2015; 13(6): 1685–93.

Goss C, Fontanesi S, Mazzi MA, Del Piccolo L, Rimondini M. The assessment of patient involvement across consultation. The Italian version of the OPTION scale. Epidemiol Psichiatr Soc 2007;16: 339–49.

Grant S, Mayo-Wilson E, Montgomery P. CONSORT-SPI 2018 Explanation and Elaboration: guidance for reporting social and psychological intervention trials. Trials 2018; 19:406.

Guillemin F, Bombardier C, Beaton D. Cross-cultural adaptation of health-related quality of life measures: literature review and proposed guidelines. J Clin Epidemiol 1993; 46:1417–1432.

Hérbert R, Bravo G, Préville M. Reliability, validity, and reference values of the Zarit Burden Interview for assessing informal caregivers of community-dwelling older persons with dementia. Canadian Journal on Aging 2000; 19: 494-507.

Herrmann C. International experiences with the Hospital Anxiety and Depression Scale--a review of validation data and clinical results. J Psychosom Res 1997; 42: 17-41.

Higginson IJ, Hart S, Silber E, Burman R, Edmonds P. Symptom prevalence and severity in people severely affected by multiple sclerosis. J Palliat Care 2006; 22: 158–65.

Hirst C, Swingler R, Compston DA, et al. Survival and causes of death in multiple sclerosis: a prospective population-based study. J Neurol Neurosurg Psychiatry 2008; 79: 1016-21.

Honarmand K, Feinstein A. Validation of the Hospital Anxiety and Depression Scale for use with multiple sclerosis patients. Mult Scler 2009;15:1518–24.

Kingwell E, van der Kop M, Zhao Y, et al. Relative mortality and survival in multiple sclerosis: findings from British Columbia, Canada. J Neurol Neurosurg Psychiatry 2012; 83: 61–6.

Kryworuchko J, Stacey D, Bennett C, Graham ID. Appraisal of primary outcome measures used in trials of patient decision support. Patient Educ Couns 2008;73: 497–503.

Kurtzke JF. Rating neurologic impairment in multiple sclerosis: an expanded disability status scale (EDSS). Neurology 1983; 33: 1444-52.

Lancaster GA, Thabane L. Guidelines for reporting non-randomised pilot and feasibility studies. Pilot and Feasibility Studies 2019; 5:114.

Lublin FD, Reingold SC. Defining the clinical course of multiple sclerosis: results of an international survey. Neurology 1996; 46: 907–11.

Lunde HMB, Assmus J, Myhr K-M, Bø L, Grytten N. Survival and cause of death in multiple sclerosis: a 60-year longitudinal population study. J Neurol Neurosurg Psychiatry 2017; 88: 621–5.

McCurry MK. An exploratory study of decision making by informal caregivers of individuals with multiple sclerosis. J Neurosci Nurs 2013; 45(1): 52–60.

Mahoney FI, Barthel D. Functional evaluation: The Barthel Index. Maryland State Med Journal 1965; 14:56-61.

May CR, Cummings A, Girling M, et al. Using Normalization Process Theory in feasibility studies and process evaluations of complex healthcare interventions: a systematic review. Implementation science 2018;13(1):80.

Moore G, Audrey S, Barker M, et al. Process evaluation of complex interventions: Medical Research Council guidance. London: MRC Population Health Science Research Network; 2014. 7.

Murray E, Treweek S, Pope C, et al. Normalisation process theory: a framework for developing, evaluating and implementing complex interventions. BMC Med. 2010;8(1):63.

O'Brien BC, Harris IB, Beckman TJ, Reed DA, Cook DA. Standards for reporting qualitative research: a synthesis of recommendations. Acad Med. 2014; 89(9): 1245–51.

Puhan MA, Frey M, Büchi S, Schünemann HJ. The minimal important difference of the hospital anxiety and depression scale in patients with chronic obstructive pulmonary disease. Health and Quality of Life Outcomes 2008, 6:46.

Rietjens JAC, Sudore RL, Connolly M, et al. Definition and recommendations for advance care planning: an international consensus supported by the European Association for Palliative Care. Lancet Oncol 2017; 18(9): e543-e551.

Rosato R, Testa S, Bertolotto A, et al. eMSQOL-29: Prospective validation of the abbreviated, electronic version of MSQOL-54. Mult Scler 2019;25(6): 856-66.

Rosato R, Testa S, Bertolotto A, et al. Development of a Short Version of MSQOL-54 Using Factor Analysis and Item Response Theory. PLoS One 2016;11(4): e0153466.

Scalfari A, Knappertz V, Cutter G, et al. Mortality in patients with multiple sclerosis. Neurology 2013; 81: 184–92.

Schichtel M, Wee B, Perera R. Clinician-targeted interventions to improve advance care planning in heart failure: a systematic review and meta-analysis. Heart 2019; 105: 1316–24.

Schickedanz AD, Schillinger D, Landefeld CS, Knight SJ, Williams BA, Sudore RL. A clinical framework for improving the advance care planning process: start with patients' self-identified barriers. J Am Geriatr Soc. 2009; 57:31–9.

Solari A, Giordano A, Kasper J, et al. Role Preferences of People with Multiple Sclerosis: Image-Revised, Computerized Self-Administered Version of the Control Preference Scale. PLoS One 2013;8:e66127.

Solari A, Giordano A, Sastre-Garriga J, et al. EAN guideline on palliative care of people with severe, progressive multiple sclerosis. Eur J Neurol. 2020;27(8):1510-29.

Sudore RL, Heyland DK, Barnes DE, et al. Measuring advance care planning: Optimizing the Advance Care Planning Engagement Survey. J Pain Symptom Manage. 2017;53(4):669-681.

Sumelahti ML, Hakama M, Elovaara I, et al. Causes of death among patients with multiple sclerosis. Mult Scler 2010; 16: 1437-42.

Tong A, Sainsbury P, Craig J. Consolidated criteria for reporting qualitative research (COREQ): a 32 item checklist for interviews and focus groups. Int J Qual Health Care. 2007; 19(6): 349–57.

Walter HAW, Seeber AA, Willems DL, de Visser M. The role of palliative care in chronic progressive neurological diseases-a survey amongst neurologists in the Netherlands. Front Neurol 2019; 14; 9: 1157.

Washington AE, Lipstein SH. The patient-centered outcomes research institute-promoting better information, decisions, and health. N Engl J Med 2011; 365: e31.

Wild D, Eremenco S, Mear I, et al. Multinational trials-recommendations on the translations required, approaches to using the same language in different countries, and the approaches to support pooling the data: the ISPOR Patient-Reported Outcomes Translation and Linguistic Validation Good Research Practices Task Force report. Value Health 2009;12(4):430-40.

Zigmond AS, Snaith RP. The Hospital Anxiety and Depression Scale. Acta Psychiatr Scand 1983; 67: 361–70.

14 GLOSSARY OF ABBREVIATIONS

AD	Advance Directive
ACP	Advance Care Planning
4-item ACP-E	4-item ACP Engagement questionnaire
BI	Barthel Index
CME	Continuing Medical Education
CPS	Control Preference Scale
DMAC	Data Management and Analysis Committee
DSMC	Data Safety and Monitoring Committee
EC	Ethics Committee
EDSS	Expanded Disability Status Scale
FGM	Focus Group Meeting
ICH	International Council for Harmonisation of Technical Requirements for Pharmaceuticals for Human Use
GCP	Good Clinical Practice
HADS	Hospital Anxiety and Depression Scale
MRC	Medical Research Council
MS	Multiple Sclerosis
MSQOL-29	29-item MS Quality of Life
NPT	Normalization Process Theory
OPTION	Observing Patient Involvement in SDM scale
PI	Principal Investigator
PwPMS	People with Progressive Multiple Sclerosis
QAP	Quality Analysis Panel
QOC	Quality of Communication questionnaire
TSC	Trial Steering Committee
ZBI	22-item Zarit Burden Interview

15 SIGNATURE PAGE

ConCure-SM Study Phase 2 Principal Investigators:

Dr. Alessandra Solari	______________________	March 15th, 2021
Unit of Neuroepidemiology	Signature	Date
Fondazione IRCCS		
Istituto Neurologico C. Besta		
Milan - Italy		

Dr. Ludovica De Panfilis	______________________	________________
Unit of Bioethics	Signature	Date
Azienda USL-IRCCS di Reggio Emilia		
Reggio Emilia - Italy		

**Sponsor's Representative**	______________________	________________
Fondazione IRCCS	Signature	Date
Istituto Neurologico C. Besta		
Milan - Italy		

**Center's Principal Investigator**	______________________	________________
	Signature	Date
