# Supplementary material for: Advance care planning in multiple sclerosis (ConCure-SM): A multicenter single-arm pilot and feasibility study
Source: PLoS One. 2025 Oct 7;20(10):e0331220. doi: 10.1371/journal.pone.0331220 (PMC12503263; doi:10.1371/journal.pone.0331220)
Supplement: S3 File — (PDF) [file pone.0331220.s003.pdf]

File S3. Interview and focus group guides.

Code |___|___|___|

Patient Interview Guide

Acknowledgments and **introduction** by the interviewer.
Explanation of the **reason for the interview**: *"You have participated in the ConCure-SM study, on ACP for people with MS. We would now like to know how was your experience of participating in the study, to deepen the positive and negative elements and the sensations experienced during the ACP process.*
We invite you to ***freely express*** *your thoughts because what you will tell us is valuable for improving the way the ACP is planned and implemented in people with MS. In order not to overlook any detail, our interview is recorded, however we remind you that the data collected will be managed in a completely confidential and anonymous form".*

Topic 1 – Presentation of the study
I ask you to think about when you were asked to participate in the study.
-Were the objectives clear to you?
-Can you tell us how you felt?
-Are there any emotions, thoughts, reflections related to the study presentation?

Theme 2 – The ACP conversation
Now I ask you to think about the ACP conversation(s).
-Did it correspond to your expectations?
-Are there positive or negative elements that you would like to share?
-Can you tell us how you felt?
-Would you like to tell your experience by sharing your emotions, thoughts and reflections?

Theme 3 – Professionals
Now let's think about the professionals involved in the conversation.
-Did any other professional participate besides your doctor?
-How do you evaluate their participation in this type of conversation?
-Can you tell us how they made you feel?
-Are there any issues (positive and negative) that you would like to share?

Topic 4 – Patient referrals
[Only if a significant other was present]. Now I ask you to think about your next to kin/loved one who participated in the conversation.
-How do you evaluate the participation of **your loved one** in the conversation?
-Are there any issues (positive and negative) that you would like to share?

ACP includes the possibility of identifying a **trustee**.
-Could you tell me what was your experience in this regard?
- How did you feel about this?
-What were your thoughts, reflections and emotions?

Theme 5 – Follow-up
-Are there any effects that the ACP has brought? [*in the relationship with your family, in the management of the disease, in the relationship with health services, in the relationship with caregivers...]*
-Have you had the opportunity to talk about it with anyone?
-Could you tell me in what situation?

Theme 6 – The supporting tool (usefulness, acceptability)
As for the booklet (designed to support and draw up the ACP)
-How did you find it?
-Are there any aspects that you would improve with respect to content and its use?
-Did you share it with your significant other outside of the conversation(s)?

Theme 7 – Research infrastructures
-Did you use the web platform dedicated to the study?
-How did you find its use? *[filling in questionnaires, email reminders...]*
-Are there any issues (positive and negative) that you would like to share?

-Have you had relations with the staff of the coordinating unit? How was your experience? *[completion of questionnaires, telephone contacts...]*
-Are there any issues (positive and negative) that you would like to share?

Conclusion and acknowledgments
Are there any reflections/thoughts you would like to share in closing?

Code |___|___|___|

Significant Other Interview Guide

Acknowledgments and **Interviewer's** Introduction
Explanation of the **reason for the interview***: "You have participated in the ConCure-SM study, on ACP for people with MS. We would now like to know how was your experience of participating in the study, to deepen the positive and negative elements and the feelings experienced during the ACP of your loved one.*
We invite you to ***freely express*** *your thoughts because what you will tell us is valuable for improving the way the ACP is planned and implemented in people with MS. In order not to overlook any detail, our interview is recorded, however we remind you that the data collected will be managed in a completely confidential and anonymous form".*

Topic 1 – Presentation of the study
I ask you to think about when you were asked to participate in the study.
-What thoughts did you have when your beloved's doctor invited you to participate in the study?
-Were the objectives clear?
-Can you tell us how you felt?
-Are there any emotions, reflections, thoughts related to the study presentation?

Theme 2 – The ACP conversation
Now I ask you to think about the ACP conversation(s).
-Did it correspond to your expectations?
-How did you feel about participating in it?
-Are there elements (positive and negative) that you would like to share?
-Would you like to tell your experience by sharing your emotions, thoughts and reflections?

Theme 3 – Professionals
Now I ask you to think about the professionals involved in the conversation.
-Did any other professional participate besides the doctor?
-How do you evaluate their participation in this type of conversation?
-Are there elements (positive and negative) that you would like to share?

Theme 4 – The Trustee
The ACP includes the possibility for the patient to identify a **trustee**.
-Could you tell me what your experience has been in this regard?
-What were your thoughts, reflections and emotions?

Theme 5 – Follow-up
-Are there any effects that the ACP has brought? [*in the relationship with your loved one, in the management of the disease, in the relationship with health services, in the relationship with caregivers...]*
-Have you had the opportunity to talk about it with anyone?

-Could you tell me in what situation?

Theme 6 – The supporting tool (usefulness, acceptability)
As for the booklet (designed to support and draw up the ACP)
-How did you find it?
-Are there any aspects that you would improve with respect to content and its use?
-Did you share it with your significant other outside of the conversation(s)?

Theme 7 – Research infrastructures
-Did you use the web platform dedicated to the study?
-How did you find its use? *[filling in questionnaires, email reminders...]*
-Are there elements (positive and negative) that you would like to share?

-Have you had relations with the staff of the coordinating unit? How was your experience? *[completion of questionnaires, telephone contacts...]*
-Are there elements (positive and negative) that you would like to share?

Conclusion and acknowledgments
Are there any reflections/thoughts you would like to share in closing?

Clinician focus group guide

Acknowledgments and explanation of the reason for the focus group.
Focus group conversation rules explained.

Theme 1 – Participation in research
Could you please tell me how do you experienced the participation in the ConCure-SM study from your point of view?
Were the objectives clear? How did you feel about participating in it?

Theme 2 – COHERENCE OF THE INTERVENTION
[The extent to which individuals truly understand all elements of ACP and the reasons for adopting a new strategy]
How did you find the intervention (training programme and use of the booklet) with respect to the objectives of your work? *Yes, because... no, because…]*
Do you think the intervention is coherent with the care objectives of your Unit? [*yes, because... no, because]*

Theme 3 – Usefulness of the booklet
Did the booklet support the ACP conversation(s)?

Theme 4 – COGNITIVE PARTICIPATION
[The extent to which individuals believe in the innovation provided by the introduced practice and begin to prepare for it]
How much do you think the ACP process can be effectively carried out in the context in which you operate? What is the ACP meaning?
What are facilitators and barriers? What are the needs [training, organizational, tools] that should be met in order to implement/improve it?
[If involved in the intervention delivery] What are the aspects that you have found most difficult to implement? How could they be addressed?

Theme 5 – COLLECTIVE ACTION
[What happens when the intervention is made operational]
What has changed/will change the ACP practice? And what about the introduction of this tool?
Has there been an active involvement by colleagues of the unit/service with respect to the ACP? [*yes, because… no, because]* What are the professional skills/actions that should be envisaged to put ACP in practice?

Theme 6 - REFLECTIVE MONITORING
[The act of monitoring an innovation and intelligently adapting it to changed/changing circumstances]
What have been the effects of implementing the ACP in your individual practice? *[Has anything changed? What did ACP leave you? Was it worth?]*
What were the effects of the ACP implementation in your Unit? *[Has anything changed? What did ACP leave? Was it worth?]*
Are there any components to change/revise to improve the ACP process?

Conclusion and acknowledgements

In summary, what are the most important indications/suggestions to spread/consolidate the proposed ConCure-SM ACP process?
