# Supplementary material for: Advance care planning in multiple sclerosis (ConCure-SM): A multicenter single-arm pilot and feasibility study
Source: PLoS One. 2025 Oct 7;20(10):e0331220. doi: 10.1371/journal.pone.0331220 (PMC12503263; doi:10.1371/journal.pone.0331220)
Supplement: S4 File — (PDF) [file pone.0331220.s004.pdf]

File S4

Consolidated criteria for reporting qualitative studies (COREQ):32-item checklist

**No. Item**	**Guide questions/description**	**Reported on Section #**
**Domain 1: Research team and reflexivity**		
Personal Characteristics		
1. Interviewer/facilitator	Which author/s conducted the interview or focus group?	5 interviews were held by LDP, 4 by RMZ.<br>LDP and SV moderated the focus group
2. Credentials	What were the researcher's credentials? E.g. PhD, MD	LDP: BSc, PhD<br>LG: BSc, PhD<br>MP: BSc, PhD student<br>RMZ: BSc, PhD<br>SV: MD, PhD
3. Occupation	What was their occupation at thetime of the study?	LDP: bioethicist researcher<br>LG: qualitative methods researcher<br>MP: bioethicist, PhD student<br>SV: palliative care physician and researcher<br>RMZ: philosophy of language researcher
4. Gender	Was the researcher male orfemale?	Three were women (RMZ, LDP, MP), and two men (LG, SV)
5. Experience and training	What experience or training didthe researcher have?	All the researchers had expertise in planning, conducting, and analyzing qualitative studies
Relationship with participants		
6. Relationship established	Was a relationship established prior to study commencement?	No relationship established prior to study commencement
7. Participant knowledge of theinterviewer	What did the participants know about the researcher? e.g. personal goals, reasons for doing the research	No knowledge
8. Interviewer characteristics	What characteristics were reported about the inter viewer/facilitator? e.g. Bias, assumptions, reasons and interests in the research topic	LDP was co-PI of the study. LDP and SV contributed to protocol devise, and they were members of the study's Steering Committee. LG, RMZ, and MP were part of the Qualitative Analysis Panel.
**Domain 2: study design**		
Theoretical framework		
9. Methodological orientation and Theory	What methodological orientation was stated to underpin the study? e.g. grounded theory, discourse analysis, ethnography, phenomenology, content analysis	The interviews and focus groups were analyzed using thematic analysis, with interpretation guided by the Normalization Process Theory (Methods – Qualitative data)
Participant selection		

10. Sampling	How were participants selected? e.g. purposive, convenience, consecutive, snowball	Interviews - A purposive sampling technique was used to select the participants<br><br>Focus groups - All clinicians involved in the intervention delivery were invited to take part in the focus groups.
11. Method of approach	How were participants approached? e.g. face-to-face, telephone, mail, email	By phone and email (Methods –Nested qualitative study)
12. Sample size	How many participants were in the study?	5 persons with progressive multiple sclerosis (PwPMS), 4 significant others (SOs), and 14 clinicians (Results-Qualitative findings and process evaluation, and Table S3)
13. Non-participation	How many people refused to participate or dropped out? Reasons?	1/6 invited PwPMS refused participation after a telephone scam<br>1/5 invited SO refused participation (unknown reason)
Setting		
14. Setting of data collection	Where was the data collected? e.g. home, clinic, workplace	Online (Methods – Nested qualitative study).
15. Presence of non-participants	Was anyone else present besides the participants and researchers?	No
16. Description of sample	What are the important characteristics of the sample? e.g. demographic data, date	Table S3 (PwPMS and SOs)<br>Table S4 (clinicians)
Data collection		
17. Interview guide	Were questions, prompts, guides provided by the authors? Was it pilot tested?	Interview and focus group guides are provided in File S2. The interview guide wasn't pilot tested.
18. Repeat interviews	Were repeat interviews carried out? If yes, how many?	Not applicable.
19. Audio/visual recording	Did the research use audio or visual recording to collect the data?	Interviews and the focus group were audio-recorded (Methods – Qualitative data).
20. Field notes	Were field notes made during and/or after the interview or focus group?	No
21. Duration	What was the duration of the interviews or focus group?	The interviews lasted between 12 minutes and 39 minutes (median 18 minutes).<br>The first focus group lasted 118 minutes, the second 106 minutes.
22. Data saturation	Was data saturation discussed?	No
23. Transcripts returned	Were transcripts returned to participants for comment and/or correction?	Yes (focus groups)
**Domain 3: analysis and findings**		
Data analysis		

24. Number of data coders	How many data coders coded the data?	Four data coders were involved: LDP, LG, MP, RMZ, SV (Methods – Qualitative data analysis)
25. Description of the coding tree	Did authors provide a description of the coding tree?	No
26. Derivation of themes	Were themes identified in advance or derived from the data?	Both inductive and deductive methods applied
27. Software	What software, if applicable, was used to manage the data?	Not applicable
28. Participant checking	Did participants provide feedback on the findings?	No
Reporting		
29. Quotations presented	Were participant quotations presented to illustrate the themes/findings? Was each quotation identified? e.g. participant number	Quotations from the interviews are reported in Table 4.
30. Data and findings consistent	Was there consistency between thedata presented and the findings?	Yes (as study authors, we can be biased in the evaluation of this domain)
31. Clarity of major themes	Were major themes clearly presented in the findings?	Yes (as study authors, we can be biased in the evaluation of this domain)
32. Clarity of minor themes	Is there a description of diverse cases or discussion of minor themes?	Not applicable
