# Supplementary material for: Advance care planning in multiple sclerosis (ConCure-SM): A multicenter single-arm pilot and feasibility study
Source: PLoS One. 2025 Oct 7;20(10):e0331220. doi: 10.1371/journal.pone.0331220 (PMC12503263; doi:10.1371/journal.pone.0331220)
Supplement: S1 Box — (PDF) [file pone.0331220.s005.pdf]

S1 Box. Conditions that would make advance care planning (ACP) relevant.

1. Express desire for ACP
2. Raise questions about person with progressive multiple sclerosis's future
3. Talk about hastening death or medically assisted suicide
4. High risk for death within two years using the '*Surprise Question*'
5. High risk for development of severe cognitive compromise/dementia within two years
6. High risk for development of impairments preventing communication within two years
7. Significant suffering (e.g., uncontrolled physical symptoms, psychosocial or existential issues)
