# Supplementary material for: Advance care planning in multiple sclerosis (ConCure-SM): A multicenter single-arm pilot and feasibility study
Source: PLoS One. 2025 Oct 7;20(10):e0331220. doi: 10.1371/journal.pone.0331220 (PMC12503263; doi:10.1371/journal.pone.0331220)
Supplement: S1 Table — (PDF) [file pone.0331220.s006.pdf]

S1 Table. Characteristics of subjects at screening across centers.

Characteristic	Overall (N=164)	Verona (N=26)	Moncrivello (N=16)	Reggio Emilia (N=13)	Bologna (N=51)	Roma (N=14)	Catania (N=44)
				N (%)			
Women	96 (58)	15 (58)	12 (75)	4 (31)	35 (69)	6 (43)	24 (55)
Age (years)[†]	58.6 (10.8)	59.0 (9.6)	50.6 (12.4)	58.6 (9.1)	61.0 (11.8)	59.6 (10.0)	58.4 (9.5)
Education							
Primary (5–8 years)	58 (35)	5 (19)	11 (69)	1 (8)	21 (43)	1 (7)	18 (41)
Secondary (12–13 years)	80 (49)	14 (54)	4 (25)	8 (61)	19 (37)	11 (79)	24 (55)
College/University (14+ years)	26 (16)	7 (27)	1 (6)	4 (31)	10 (20)	2 (14)	2 (4)

[†1] Mean (standard deviation).
