# Supplementary material for: Advance care planning in multiple sclerosis (ConCure-SM): A multicenter single-arm pilot and feasibility study
Source: PLoS One. 2025 Oct 7;20(10):e0331220. doi: 10.1371/journal.pone.0331220 (PMC12503263; doi:10.1371/journal.pone.0331220)
Supplement: S2 Table — (PDF) [file pone.0331220.s007.pdf]

S2 Table. Participants' characteristics at baseline across centers. ACP, advance care planning; EDSS, Expanded Disability Status Scale; HADS, Hospital Anxiety and Depression Scale; MSQOL-29, Multiple Sclerosis Quality of Life-29 items; MHC, mental health composite; MS, multiple sclerosis; NA, not applicable; PHC, physical health composite; ZBI, Zarit Burden Interview.

**Characteristic**	**Overall (n=19)**	**Verona (n=3)**	**Moncrivello (n=3)**	**Reggio Emilia (n=3)**	**Bologna (n=2)**	**Rome (n=5)**	**Catania (n=3)**
				N (%)			
People with progressive multiple sclerosis							
Women	8 (42)	1 (34)	2 (67)	1 (34)	0	2 (40)	2 (67)
Age (years)†	61.6 (7.6)	60.0 (9.5)	57.3 (5.5)	63.3 (8.4)	54.0 (1.4)	63.2 (4.9)	68.0 (10.8)
Education:							
Primary (5-8 years)	2 (10)	0 (0)	1 (33)	0 (0)	0 (0)	1 (20)	0 (0)
Secondary (12-13 years)	14 (74)	3 (100)	2 (67)	1 (33)	1 (50)	4 (80)	3 (100)
College/University (14+ years)	3 (16)	0 (0)	0 (0)	2 (67)	1 (50)	0 (0)	0 (0)
Occupation:							
Retired (disability)	11 (58)	3 (100)	2 (67)	1 (33)	1 (50)	4 (80)	0 (0)
Employed	4 (21)	0 (0)	1 (33)	1 (33)	1 (50)	0 (0)	1 (33)
Retired (age)	3 (16)	0 (0)	0 (0)	1 (34)	0 (0)	0 (0)	2 (67)
Housewife	1 (5)	0 (0)	0 (0)	0 (0)	0 (0)	1 (20)	0 (0)
Age at first MS symptoms (years)†	37.4 (12.0)	28.3 (3.8)	45.6 (2.1)	37.7 (15.7)	24.0 (5.7)	38.4 (12.1)	45.3 (15.0)
Age at MS diagnosis (years)†	40.3 (14.4)	29.0 (3.5)	48.7 (2.1)	43.0 (19.3)	16.0 (7.1)	42.8 (5.0)	52.3 (16.3)
EDSS‡	8.0 (6.5-8.0)	8.0 (8.0-8.0)	8.0 (7.25-8.0)	5.0 (5.0-5.75)	8.0 (7.5-8.5)	8.0 (8.0-8.5)	8.0 (7.25-8.0)
MS course:							
Primary progressive	8 (42)	1 (33)	2 (67)	1 (33)	1 (50)	2 (40)	1 (33)
Secondary progressive	11 (58)	2 (67)	1 (33)	2 (67)	1 (50)	2 (60)	2 (67)
Barthel index†	36.7 (28.5)	38.3 (22.5)	42.3 (24.8)	74.3 (22.4)	25.0 (29.7)	21.8 (15.7)	37.0 (44.2)
HADS Anxiety†,§	4.7 (3.8)	4.3 (1.1)	9.3 (7.0)	5.0 (5.7)	5.5 (2.1)	2.4 (1.5)	3.3 (1.1)
HADS Depression†,§	5.7 (5.1)	1.0 (0)	10.0 (8.9)	4.0 (4.2)	8.5 (3.5)	3.0 (1.6)	10.0 (2.7)
4-item ACP-Engagement†,§	2.9 (1.1)	3.8 (0.7)	2.8 (1.1)	2.9 (0.2)	2.9 (1.9)	2.6 (1.4)	2.8 (1.3)

MSQOL-29 PHC†,§	36.0 (7.7)	38.4 (5.5)	37.7 (14.1)	37.2 (9.6)	39.5 (2.0)	34.2 (7.7)	30.2 (5.4)
MSQOL-29 MHC†,§	60.0 (11.9)	63.2 (13.8)	56.0 (10.0)	57.5 (6.7)	53.1 (7.3)	69.0 (12.0)	49.9 (12.8)
Significant others							
Women	5 (50)	1 (100)	-	-	1 (100)	2 (40)	1 (33)
Age (years)†	57.3 (10.9)	49.0 (NA)	-	-	54.0 (NA)	63.0 (8.0)	51.7 (15.4)
Education:							
Secondary (12-13 years)	7 (70)	1 (100)	-	-	1 (100)	3 (60)	2 (67)
College/university (14+ years)	3 (30)	0 (0)	-	-	0 (0)	2 (40)	1 (33)
Occupation:							
Employed	5 (50)	0 (0)	-	-	1 (100)	2 (40)	2 (67)
Retired (age)	2 (20)	0 (0)	-	-	0 (0)	2 (40)	0 (0)
Housewife	3 (30)	1 (100)	-	-	0 (0)	1 (20)	1 (33)
Relation:							
Spouse/partner	7 (70)	1 (100)	-	-	1 (100)	3 (60)	2 (67)
Other relative	2 (20)	0	-	-	0 (0)	2 (40)	0
Son/daughter	1 (10)	0	-	-	0 (0)	0	1 (33)
ZBI total score†, ¶	21.7 (11.6)	8.0 (NA)	-	-	37.0 (NA)	24.3 (10.6)	17.0 (9.9)

† Mean (standard deviation).

‡ Median (IQR)

§ One patient refused to complete questionnaire.

¶ Three significant others did not complete questionnaire.
