# Supplementary material for: Advance care planning in multiple sclerosis (ConCure-SM): A multicenter single-arm pilot and feasibility study
Source: PLoS One. 2025 Oct 7;20(10):e0331220. doi: 10.1371/journal.pone.0331220 (PMC12503263; doi:10.1371/journal.pone.0331220)
Supplement: S3 Table — (PDF) [file pone.0331220.s008.pdf]

Table S3. Per protocol analysis of the secondary outcome measures. Significant values are reported in bold. ACP, advance care planning; HADS, Hospital Anxiety and Depression Scale; IQR, interquartile range; MHC, mental health composite; MSQOL-29, 29-item Multiple Sclerosis Quality of Life; PHC, physical health composite; ZBI, Zarit Burden Interview.

**Secondary outcome measures**							
	**Baseline**		**First ACP conversation**		**6-month follow up**		**P value**
	n	*Mean, median (IQR)*	*n*	*Mean, median (IQR)*	*n*	*Mean, median (IQR)*	
**Persons with progressive multiple sclerosis†**							
HADS-Anxiety	18	4.7, 4 (2-5)	18	5.5, 6 (3-8)	17	7.1, 6 (3-10)	**0.001**
HADS-Depression	18	5.7, 4.5 (1-8)	18	6.5, 6.5 (2-9)	17	6.9, 7 (4-8)	0.25
4-item ACP-Engagement	18	11.6, 12.5 (7-15)	18	12.8, 13.5 (9-17)	17	13.1, 13 (9-20)	0.37
MSQOL-29, PHC	18	36.4, 37.5 (33-41)	-	-	19	33.5, 35.5 (24.5-40)	0.32
MSQOL-29, MHC	18	59.6, 60.9 (48.4-68.4)	-	-	18	51.4, 52.9 (44.3-64.3)	**0.01**
**Significant others†**							
ZBI total score	7	21.7, 24 (10-34)	9	23, 23 (13-30)	7	22.7, 19 (14-27)	0.59

† Generalized estimating equations.
