# Supplementary material for: Advance care planning in multiple sclerosis (ConCure-SM): A multicenter single-arm pilot and feasibility study
Source: PLoS One. 2025 Oct 7;20(10):e0331220. doi: 10.1371/journal.pone.0331220 (PMC12503263; doi:10.1371/journal.pone.0331220)
Supplement: S4 Table — (PDF) [file pone.0331220.s009.pdf]

Table S4. Normalisation MeAsure Development questionnaire (NoMAD) scale scores of 13 clinicians (7 neurologists, 6 other professionals) from the six participating centers.

Score (0-100)	Mean (SD)	95% CI	Range
Sense making (coherence)	91.1 (5.1)	88.1–94.2	85-100
Cognitive participation	89.2 (9.3)	83.6–94.9	75-100
Collective action	79.6 (11.0)	73.0–86.2	63-100
Reflexive monitoring	87.1 (10.6)	80.7–93.5	72-100
