# Supplementary material for: Advance care planning in multiple sclerosis (ConCure-SM): A multicenter single-arm pilot and feasibility study
Source: PLoS One. 2025 Oct 7;20(10):e0331220. doi: 10.1371/journal.pone.0331220 (PMC12503263; doi:10.1371/journal.pone.0331220)

S1 Fig. Scatter plots of Hospital Anxiety and Depression Scale (HADS)-Anxiety scores at baseline, after the first advance care planning (ACP) conversation (T1), and at 6-month follow-up (T2) (per-protocol data). Codes of people with progressive multiple sclerosis who completed the ACP-Document are reported in bold.

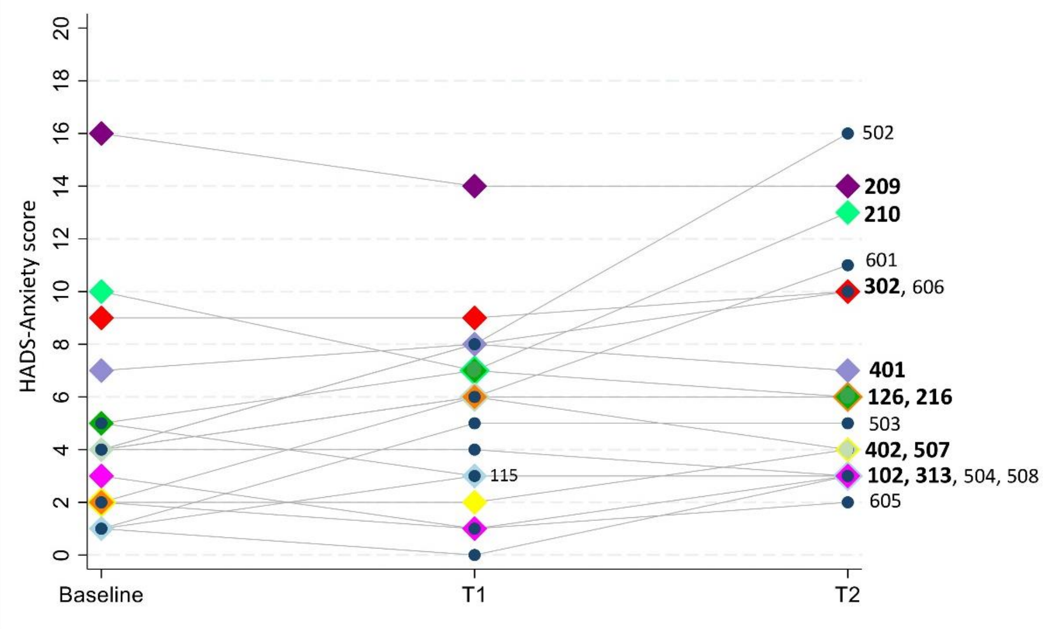

Supplement: S1 Fig — Codes of people with progressive multiple sclerosis who completed the ACP-Document are reported in bold. (PDF) [file pone.0331220.s010.pdf]
