# Supplementary material for: Advance care planning in multiple sclerosis (ConCure-SM): A multicenter single-arm pilot and feasibility study
Source: PLoS One. 2025 Oct 7;20(10):e0331220. doi: 10.1371/journal.pone.0331220 (PMC12503263; doi:10.1371/journal.pone.0331220)

S4 Fig. Box plots of MSQOL-29 Physical Health Composite (A) and MSQOL-29 Mental Health Composite scores (B) at baseline and at 6-month follow up (T2). The boxes represent the interquartile range, horizontal lines inside boxes represent medians and tails represent the 5th–25th and 75th–95th percentile range. Dots are outliers. MSQOL-29, 29-item Multiple Sclerosis Quality of Life; MHC, mental health composite; PHC, physical health composite.

(A)

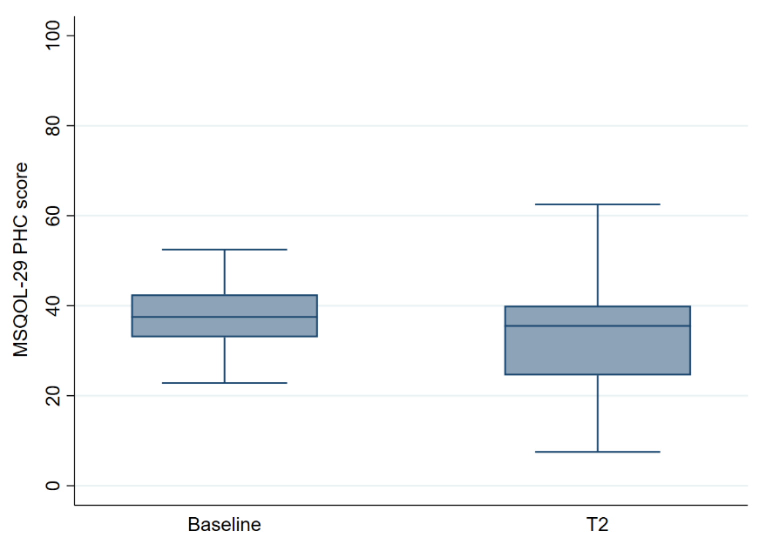

(B)

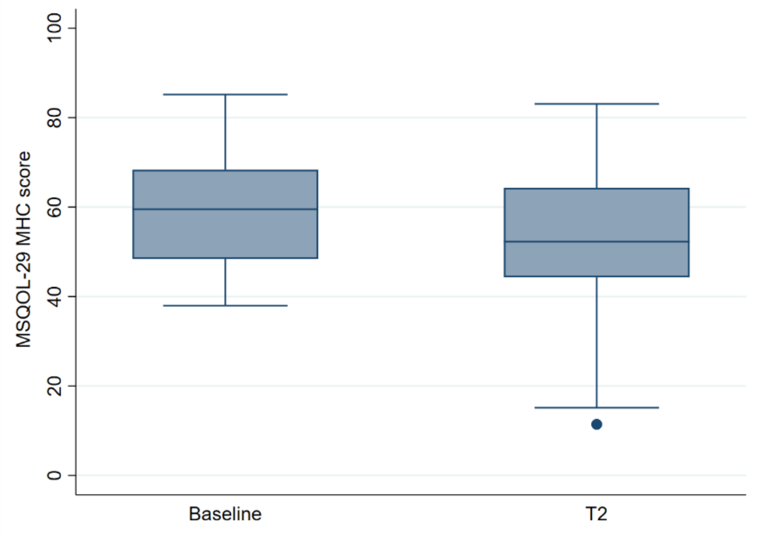

Supplement: S4 Fig — The boxes represent the interquartile range, horizontal lines inside boxes represent medians and tails represent the 5th–25th and 75th–95th percentile range. Dots are outliers. MSQOL-29, 29-item Multiple Sclerosis Quality of Life; MHC, mental health composite; PHC, physical health composite. (PDF) [file pone.0331220.s013.pdf]
