# Supplementary material for: Advance care planning in multiple sclerosis (ConCure-SM): A multicenter single-arm pilot and feasibility study
Source: PLoS One. 2025 Oct 7;20(10):e0331220. doi: 10.1371/journal.pone.0331220 (PMC12503263; doi:10.1371/journal.pone.0331220)

S5 Fig. Box plots of ZBI scores at baseline, after the first advance care planning conversation (T1), and at 6-month follow up (T2). The boxes represent the interquartile range, horizontal lines inside boxes represent medians and tails represent the 5th–25th and 75th–95th percentile range. Dots are outliers. ZBI, Zarit Burden Interview.

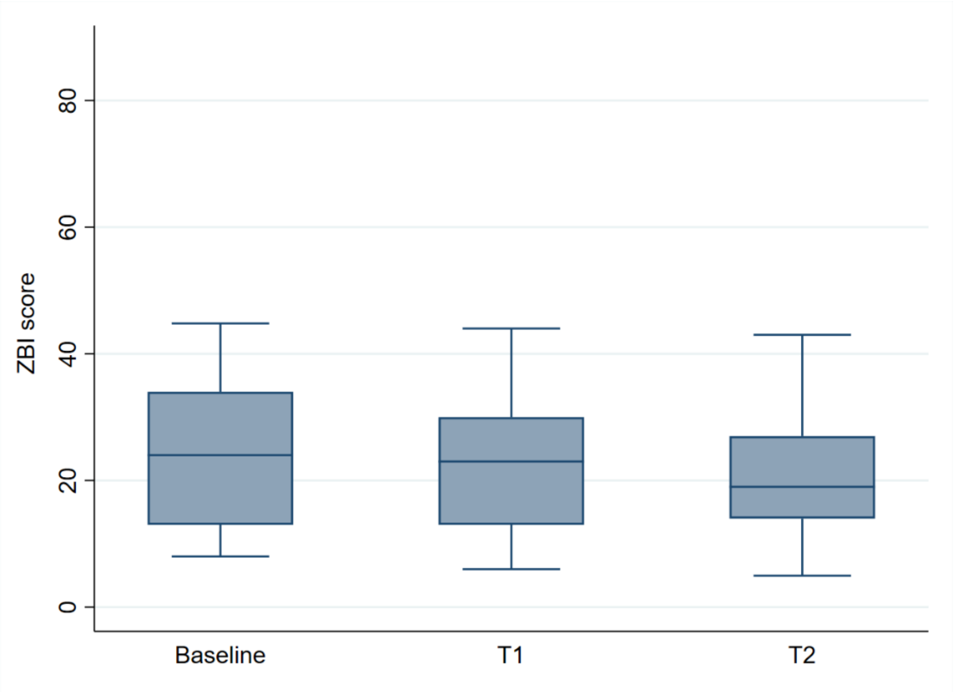

Supplement: S5 Fig — The boxes represent the interquartile range, horizontal lines inside boxes represent medians and tails represent the 5th–25th and 75th–95th percentile range. Dots are outliers. ZBI, Zarit Burden Interview. (PDF) [file pone.0331220.s014.pdf]
