# Supplementary material for: Advance care planning in multiple sclerosis (ConCure-SM): A multicenter single-arm pilot and feasibility study
Source: PLoS One. 2025 Oct 7;20(10):e0331220. doi: 10.1371/journal.pone.0331220 (PMC12503263; doi:10.1371/journal.pone.0331220)
Supplement: Attachment — Submitted filename: Response to reviewers.docx [file pone.0331220.s017.docx]

**RESPONSE TO REVIEWER #1**

*In the section of other outcome measures in the Results: These findings should be interpreted with caution (see Discussion, ‘Study limitations’ section). Please add, "due to small sample size and multiplicity problem".*

R: We have added the phrase, as suggested.
